# Viral evolution during primary infection in immunocompromised hosts

**DOI:** 10.1371/journal.pcbi.1013967

**Published:** 2026-02-25

**Authors:** Morgan Craig, Xiaoyan Deng, David V. McLeod

**Affiliations:** 1 Département de mathématiques et statistique, Université de Montréal, Montréal, Canada; 2 CHU Saint-Justine Azrieli Research Centre, Montréal, Canada; University Hospital Schleswig-Holstein - Campus Kiel: Universitatsklinikum Schleswig-Holstein, GERMANY

## Abstract

The immune response to viral infection is a delicate balance. By perturbing this balance, immunodeficiencies are expected to influence within-host viral evolution. Indeed, the presence of immunocompromised hosts has been argued to be a source of novel viral variants in some infectious diseases, including SARS-CoV-2. However, these arguments rest upon between-host models and so the role of immunodeficiencies on within-host evolution in primary infections is poorly understood. Using a mechanistic immunological model, here we consider how different immunodeficiencies shape the orchestration of the immune response during primary infection. We study how this alters the viral fitness landscape, thus speeding and slowing viral evolution. We show that during acute infections, while immunodeficiencies in neutrophils and interferon initially speed viral evolution, by the time the infection is cleared, mutations are at lower frequencies than in immunocompetent hosts. In persistent infections, we show that while T cell deficiencies slow viral evolution, interleukin-6 and macrophage deficiencies speed viral evolution. Finally, we show that positive epistatic interactions arising due to the immunological response will accelerate the evolution of viral mutations affecting the ability of virions to evade different aspects of the immune response and to enter host cells.

## Author summary

A robust immune response to viral infections is key to host protection. Upon first exposure, the immune system activates an elaborate response involving innate, adaptive, and humoral immunity. An inadequate or poorly regulated immune response, as observed in immunocompromised individuals, can result in a reduced ability to clear the virus, prolonging infection and potentially altering viral evolution. Here, we use a comprehensive mechanistic model of the immune response to a primary respiratory viral infection to study how immunodeficiencies affect the speed of within-host viral evolution. Our results show when perturbing the multiple interacting networks regulating the immune response leads to stronger selection for specific mutations, and when it does not. Overall, our study helps to untangle the interplay between the orchestration of immune responses and viral evolution in immunocompetent and immunocompromised hosts.

## Introduction

The immune response to viral infection is tightly coordinated through a series of integrated, nonlinear networks [1]. Immunodeficiencies perturb this delicate balance, altering immunological responses and prolonging infection. In turn, immunodeficiencies alter the viral fitness landscape, influencing viral evolution and the speed of viral adaptation. Indeed, many viruses of importance for public health, including HIV [2], influenza [3,4], SARS-CoV-2 [5,6], and HSV [7], show increased mutations and frequent within-host viral evolution in immunocompromised hosts [8,9], depending on the type and severity of the immunodeficiency [10] (see, e.g., Raglow et al. [11]).

Because immunocompromised hosts may shed virus throughout the course of infection, within-host evolution creates the possibility of forward transmission of novel viral mutations or variants. For example, several SARS-CoV-2 variants have been hypothesized to have originated from accelerated evolution in immunocompromised hosts [12–15], in part due to their genetic differences from other variants circulating in the population [16]. Mathematical modelling of this hypothesis predicts that the longer duration of infection in immunocompromised hosts can speed viral evolution [17,18] relative to transmission chains of acute infections, and can produce genetically differentiated variants in the presence of fitness valleys in the viral fitness landscape [19,20].

However, by focusing on the between-host dynamics, this previous work ignores within-host processes. Mutations are treated as neutral within-host, with the fitness valley occurring because “intermediate” mutations are selected against during between-host transmission, before a “jackpot” mutation is acquired [19,20]. Recent within-host modelling, on the other hand, predicts the strength of immune pressure is critical to reproducing within-host evolutionary patterns [21]. Yet, as this work relies on statistical fitness landscapes that abstract the immunological dynamics, how the nature and severity of immunodeficiencies affect the speed of within-host viral adaptation remains poorly understood.

Here, we consider how immunodeficiencies affecting the orchestration of the immune response determine the speed of within-host viral evolution in acute and persistent infections. We show that during acute infections, immunodeficiencies in neutrophils and interferon have the largest evolutionary role: while they can speed evolution early in the infection, by the time the infection has been cleared, mutations will be at a lower frequency in immunocompromised hosts. During persistent infections, T cell concentrations play a pivotal role: T cell deficiencies slow viral evolution, while interleukin-6 and macrophage deficiencies overstimulate T cells, speeding viral evolution. We then show how immunological interactions between mutations affecting evasion of different aspects of the immune response can generate epistasis in fitness, further affecting the speed of viral evolution during persistent infections. Together, this work underlines how dysregulated immune responses impact within-host viral evolution, with potential consequences for individual and population health.

## Methods

### Mathematical model of the immune response to primary infection

**Fig 1:**
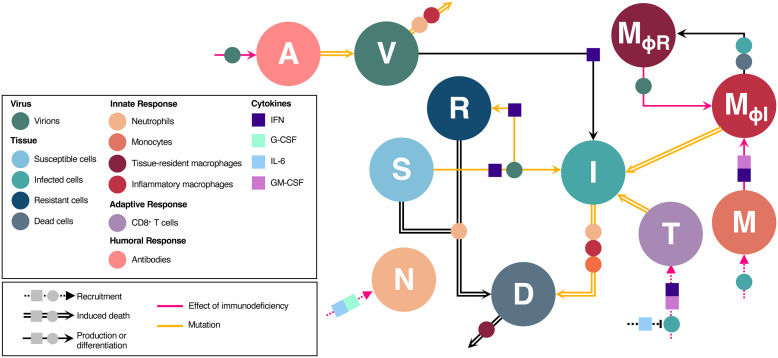
Schematic of immune response to viral infection. Virions, V, infect susceptible cells, S, producing infected cells, I. Following the eclipse phase, infected cells lyse, producing virions. Cells refractory to infection, R, are created through upregulation of interferon (IFN) signalling by infected cells. Inflammatory macrophages, $M_{\Phi I}$, are converted from monocytes, M, or tissue-resident macrophages, $M_{\Phi R}$, and along with neutrophils, N, phagocytose virions and infected cells. Phagocytosis of infected cells produces dead and damaged cells, D. Antibodies, A, are mobilized by the presence of viral antigens, and neutralize virions. CD8 + effector T cells, T, are stimulated by, and subsequently kill, infected cells. The action of the immune system is orchestrated through the antiviral cytokine IFN as well as the proinflammatory cytokines IL-6, G-CSF, and GM-CSF. Circles and boxes along arrows indicate the action of either a cell or cytokine, respectively. Dashed arrows: recruitment. Solid arrow: production or differentiation. Double arrows: induced death. Pink arrows: decreased effect due to immunodeficiency. Yellow arrows: immune effect targeted by mutations.

Our model describes the immune dynamics following a primary infection by a respiratory virus. In what follows, we highlight the key aspects of the model; complete details are provided in S1 Text, including information on parameter estimation. A model schematic is presented in Fig 1.

A primary infection occurs when virions, V(t), infect susceptible cells, S(t), to create infected cells, I(t), via mass-action at rate β. The parameter β thus captures the process of viral engulfment and cellular entry by endocytosis [22–25]. Infected cells then enter an eclipse phase lasting $\tau_{I}$ days during which they are non-productively infected. Following the eclipse phase, infected cells are productively infected and die at per-capita rate $d_{I}$, releasing p new virions (burst size) upon lysis. Virions naturally decay at a per-capita rate $d_{V}$.

Primary infection stimulates the innate, adaptive, and humoral immune responses. The innate immune response consists of three parts. First, infected cells secrete Type I interferon (IFN) at a saturable rate with a maximal secretion rate of $\varepsilon_{F,I}$. Secretion of IFN makes infected cells refractory to infection while also blocking viral entry and replication in neighbouring cells. Second, neutrophils, N(t), clear virions at per-capita rate $\delta_{V,N}$ and kill infected cells at per-capita rate $\delta_{I,N}$. Neutrophils are recruited to the site of infection through interleukin-6 (IL-6) and granulocyte colony-stimulator factor (G-CSF) cytokine signalling. Third, inflammatory macrophages, $M_{\Phi I}(t)$, destroy virions and infected cells through phagocytosis [26] at per-capita rates $\delta_{V,M}$ and $\delta_{I,M}$, respectively. Inflammatory macrophages are created from monocytes, M(t), through IL-6 and granulocyte macrophage colony-stimulator factor (GM-CSF) signalling, or from tissue-resident macrophages, $M_{\Phi R}(t)$, after contact with infected or dead, D(t), cells.

The adaptive immune response consists of CD8 + effector T cells, T(t), and is activated $\tau_{T}$ days following primary infection. CD8 + T cells are recruited by infected cells and IFN signalling and suppressed by IL-6, which serves as a proxy for anti-inflammatory signalling [27]. CD8 + effector T cells kill infected cells through density-dependent saturable killing [28] at a maximal rate of $\delta_{I,T}$.

The humoral immune response consists of antibodies, A(t), which are generated by the presence of virions after $\tau_{A}$ days. Following activation, antibodies remove virions through saturable neutralization of virions. We assume all antibodies are neutralizing, with a maximal neutralization rate of $\delta_{V,A}$, half-effect concentration $\varepsilon_{V,A}$, and Hill coefficient $h_{A}$.

Under these assumptions, the dynamics of the virions and infected cells are given by:


$$\begin{array}{c} \frac{dV}{dt}=pI(t)-\overset{m_{V}}{\overbrace{\left(d_{V}+\delta_{V,N}N(t)+\delta_{V,M}M_{\Phi ,I}(t)+\frac{{\delta_{V,A}A(t)}^{h_{A}}}{\left(\varepsilon_{V,A}^{h_{A}}+{A(t)}^{h_{A}}\right)}\right)}}V(t),\; \end{array}$$ (1)

$$\begin{array}{c} \frac{dI}{dt}=\underset{B_{I}}{\underbrace{\frac{\beta S\left(t-\tau_{I}\right)\epsilon_{F,I}}{\epsilon_{F,I}+F_{b}(t)}}}V\left(t-\tau_{I}\right)-\underset{m_{I}}{\underbrace{\left(d_{I}+\frac{\delta_{I,N}{N(t)}^{h_{N}}}{IC_{\text{5}\text{0},N}^{h_{N}}+{N(t)}^{h_{N}}}+\delta_{I,M}M_{\Phi ,I}(t)+\frac{\delta_{I,T}T(t)}{\varepsilon_{\delta ,T}+{I(t)}^{h_{T}}}\right)}}I(t), \end{array}$$ (2)

where $F_{b}(t)$ is the concentration of bound IFN at time t. In equations (1) and (2), $m_{V}$ and $m_{I}$ denote the per-capita loss of virions and infected cells, respectively, and $B_{I}$ denotes the per-capita production of infected cells by virions.

### Modelling immunodeficiencies

Immunocompromised hosts may experience protracted “acute” (i.e., persistent) infection leading to a loss of IFN effects on refractory cells [29–32], as well as a deficient innate, adaptive, and/or humoral immune response [33]. To capture the loss of IFN effects on refractory cells, we suppose they revert to susceptibility at per capita rate $\delta_{R}$ after $\tau_{R}$ days (see S1 Text Section 1). To capture immunodeficiencies, we reduce the rate of production, and hence concentration, of different state variables of the immune response. For example, a monocyte production deficiency translates to a lower concentration of monocytes at homeostasis and throughout the course of the infection. The interconnectedness of the immune response means that production deficiencies in one variable may also impact other variables (e.g., the cytokine G-CSF recruits neutrophils, and so a G-CSF deficiency will also lead to a reduction in neutrophil concentrations). Therefore, for each immunodeficiency we first establish homeostasis for all model variables at the specified level of production deficiency prior to infection (see S1 Text Section 3 for more details).

Previous studies have shown immunodeficiencies lead to aberrant production and/or generation rates of different aspects of the immune response [34], with cascading effects to other cell types [35]. We therefore assume immunodeficiencies reduce the target variable by at least 50% compared to healthy hosts. To mimic clinically observed T cell immunodeficiencies, such as advanced HIV disease defined by CD4 + T cell counts <200 cells/μl [36], a T cell production deficiency is assumed to reduce the production of T cells by 75%. To capture the near complete lack of antibodies in certain primary B cell immunodeficiencies [37], including B cell lymphomas [8], antibody immunodeficiencies are assumed to reduce antibody production by 90%. For all other state variables, to achieve at least a 50% reduction in state variable concentration, we reduce production rates by 35% (S1 Text Section 3). In all cases, varying the strength of the immunodeficiency quantitatively affects the reduction in the target variable and the magnitude of concomitant changes in the non-target variables, but does not qualitatively affect our results (Fig A in S1 Text).

### Modelling viral evolution

As our interest is how immunodeficiencies affect the strength of selection on viral evasion of the immune response, we ignore *de novo* mutations and assume that any viral mutation(s) of interest are present at a low frequency of 0.01 in the initial viral inoculum. Each viral mutation we consider targets evasion of a single immunological variable (e.g., neutrophils) in a particular life history stage (i.e., virion or infected cell). We refer to the immunological variable directly targeted by the viral mutation as the “target” and the other immunological variables as “nontargets”. Each viral mutation is beneficial, and for simplicity, cost-free for the virus (the addition of costs is a straightforward extension).

We considered mutations to be divided into two groups based on the life history stage whose fitness they directly benefit. In the first group are mutations that directly increase a component of virion fitness (henceforth, *virion mutations*). Virion mutations target virion evasion of neutrophils (decreased $\delta_{V,N}$), macrophages (decreased $\delta_{V,M}$), or antibodies (decreased $\delta_{V,A}$), or target virion infection of susceptible cells, by increasing virion host cell entry [38,39] (increased β), or by decreasing the ability of interferon to block viral entry [40,41] (increased $\varepsilon_{F,I}$). In the second group are mutations that directly increase a component of infected cell fitness (henceforth, *infected cell mutations*). Infected cell mutations target infected cell evasion of neutrophils (decreased $\delta_{I,N}$), macrophage (decreased $\delta_{I,M}$), or T cells (decreased $\delta_{I,T}$).

## Results

### The speed of within-host evolution of individual mutations

To study how the immunological environment determines the speed of evolution of a single segregating mutation, note that the frequency of mutation x within the viral population, $p_{x}(t)$, changes according to the equation


$$\begin{array}{c} \frac{dp_{x}}{dt}=s_{x}(t)p_{x}\left(\text{1}-p_{x}\right), \end{array}$$ (3)

where $s_{x}(t)$ is the time-varying selection coefficient. Thus, the strength of selection on mutation x at time t is determined by the magnitude of $s_{x}(t)$, which dictates the instantaneous rate of increase of the frequency of a mutation due to selection. The time-averaged strength of selection on mutation x from time $t_{\text{0}}$ to time t is given by


$$\begin{array}{c} \left\langle s_{x}(t)\right\rangle =\frac{\text{1}}{t-t_{\text{0}}}\int_{t_{\text{0}}}^{t}s_{x}(\tau )d\tau . \end{array}$$ (4)

Equation (4) measures how strong constant selection would have to be to yield the observed change in mutation frequency by time t. Thus, $s_{x}(t)$ and $\left\langle s_{x}(t)\right\rangle$ capture how the immune response determines the short- and long-term strength of selection, respectively, dictating the speed of adaptation. Because the strength of selection depends on the size of the mutational effect, in our figures we will normalize the selection coefficients and the time-averaged strength of selection by their maximum values over the duration of infection (e.g., $s_{x}(t)/\text{max}s_{x}(t)$).

Calculating selection coefficients in populations with different life history stages (e.g., virions and infected cells) is not trivial, particularly for populations with temporally varying per-capita growth rates [42–45]. Therefore, we first approximate $s_{x}(t)$ from simulation data as


$$\begin{array}{c} s_{x}(t)\;\approx -\frac{\text{1}}{t_{i}-t_{i-\text{1}}}\text{ln}\left(\frac{p_{x}^{I}\left(t_{i-\text{1}}\right)\left(\text{1}-p_{x}^{I}\left(t_{i}\right)\right)}{p_{x}^{I}\left(t_{i}\right)\left(\text{1}-p_{x}^{I}\left(t_{i-\text{1}}\right)\right)}\right), \end{array}$$ (5)

where $p_{x}^{I}(t)$ is the frequency of mutation x at time t in infected cells and $t_{i}-t_{i-\text{1}}$ is some (short) increment of time (e.g., hours, days); $\left\langle s_{x}(t)\right\rangle$ can be approximated using equation (5) by setting $t_{i-\text{1}}=t_{\text{0}}$. Although equation (5) only considers the frequency of the mutation in infected cells, simulation results yield similar predictions if equation (5) is calculated using the frequency of the mutation in virions (Fig B in S1 Text). The principal area of divergence between the different approximations of selection occurs at the outset of the infection: because each infection starts with only virions present, the delay in production of infected cells due to the eclipse phase means equation (5) cannot be accurately calculated during the initial few hours of the infection (Fig B in S1 Text). Therefore, in all simulation results we plot equation (5) from hour 12 onwards.

Second, if we suppose mutation x is of weak phenotypic effect and that the immunological variables and density of susceptible cells change slowly relative to the change in infected cells and virions (e.g., see Day et al. [42]), we can analytically approximate $s_{x}(t)$ as


$$\begin{array}{c} s_{x}(t)\approx \left(\frac{\text{1}}{\text{2}}\left(\text{1}-\frac{\;\overset{―}{m}}{\theta }\right)\frac{\partial m_{V}}{\partial x}+\frac{\text{1}}{\text{2}}\left(\text{1}+\frac{\overset{―}{m}}{\theta }\right)\frac{\partial m_{I}}{\partial x}+\frac{pB_{I}}{\theta }\frac{\partial f}{\partial x}\right)|\Delta x|+𝒪\left(\Delta x^{\text{2}}\right), \end{array}$$ (6)

where |Δx| is the (small) phenotypic difference between mutant and wildtype, $\overset{―}{m}\equiv m_{V}-m_{I}$, $\theta \equiv \sqrt{\text{4}pB_{I}+{\overset{―}{m}}^{\text{2}}}$, $B_{I}$ is evaluated when $\tau_{I}=\text{0}$, and ∂f/∂x=1 if x = β and $\partial f/\partial x=F_{b}/\left(\varepsilon_{F,I}+F_{b}\right)$ if $x=\varepsilon_{F,I}$ (Sup. Info. Section 4). Equation (6) reveals that selection on each mutation is the direct effect of mutation x on the viral fitness component $z\in \{m_{V},m_{I},f\}$, captured by $\frac{\partial z}{\partial x}|\Delta x|$ and weighted by the frequency of the mutation in the different life history stages (virions and infected cells) as well as the relative value of each life history stage for their contribution to future generations (reproductive value [42]). Since each mutation is beneficial and has a single effect on virion life history, only one of the direct effects in equation (6) will be nonzero and positive.

As the per-capita rate of virion destruction is expected to be higher than that of infected cells (i.e., $\overset{―}{m}>\text{0}$), a decrease in $B_{I}\;$ and/or $m_{I}$ decreases the value of virion mutations and increases the value of infected cell mutations, whereas a decrease in $m_{V}$ has the opposite effect. This has important consequences for the strength of selection.

#### Availability of susceptible cells, and reversion to susceptibility of refractory cells, determines dynamical phases of primary infections.

**Fig 2:**
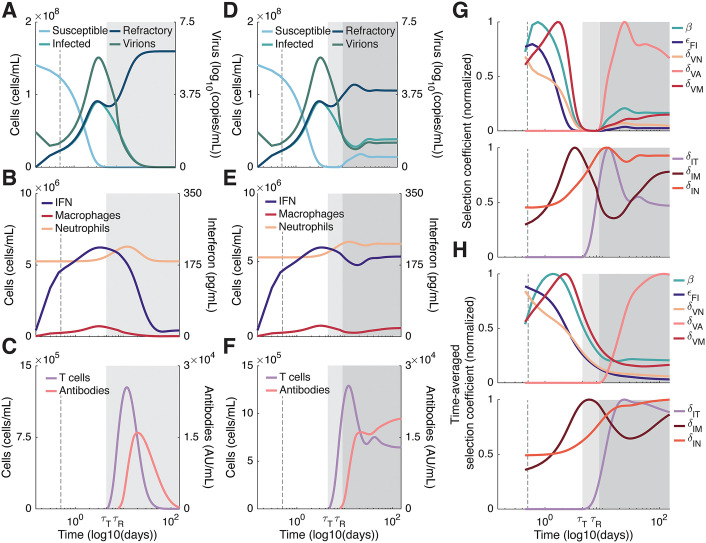
Immune dynamics distinguish the phases of infection. **A-C)** In immunocompetent hosts, the infection can be divided into two phases. In the first phase (white shaded backgrounds), susceptible cells are abundant while the immune response is mobilizing. In the second phase (light grey shaded backgrounds), susceptible cells are depleted, and the immune response eliminates the infection. **D-F)** The reversion to susceptibility of refractory cells ($\delta_{R}>\text{0}$) leads to a third infection phase (dark grey shaded backgrounds) in immunocompromised hosts. This timescale is consistent with observations of persistent infections lasting months [8,9,47]. **G-H)** In both immunocompetent and immunocompromised hosts, the abundance of susceptible cells during the first phase of infection means the adaptive value of virion mutations (i.e., mutations affecting cell entry β, as well as virion evasion of IFN, $\varepsilon_{F,I}$, neutrophils, $\delta_{V,N}$, macrophage, $\delta_{V,M}$, and antibodies, $\delta_{V,A}$) is maximized, while the value of infected cell mutations (i.e., mutations affecting infected cell evasion of neutrophils, $\delta_{I,N}$, macrophage, $\delta_{I,M}$, and T cells, $\delta_{I,T}$) is minimized. At the same time, the mobilizing immune response (**B** and **E**) increases the direct effect of selection for immune evasion. During the second phase, the absence of susceptible cells means virion mutations are selectively neutral, while the adaptive value of infected cell mutations increases. This causes a rebound in the value of virion mutations, and a decrease in the value of infected cell mutations. The mobilizing adaptive and humoral response (**C** and **F**) selects for T cell and antibody evasion. **D-F)** No additional immunodeficiencies other than $\delta_{R}>\text{0}$ are considered. **A-F)** White background: first phase of infection. Light grey background: second phase of infection. Dark grey background: third phase of infection. Dashed vertical grey line: 12 hours post-infection (see Fig B in S1 Text).

The availability of susceptible cells, and the reversion to susceptibility of refractory cells, partitions the infection into three phases exhibiting qualitatively distinct dynamics (Fig 2). In the first phase of infection, susceptible cells are abundant and the infection grows rapidly while the innate response (neutrophils, IFN, and macrophage) mobilizes (Fig 2A and 2B). As susceptible cells are abundant, the ratios $-\overset{―}{m}/\theta$ and ${pB}_{I}/\theta$ are maximized. Thus, the value of virion mutations is at its maximum while the value of infected cell mutations is at its minimum (equation (6); Fig 2G and 2H).

In the second phase of infection, susceptible cells are largely depleted, and the innate immune response is fully mobilized. Because susceptible cells are depleted, $B_{I}\to \text{0}$, and equation (6) reduces to


$$\begin{array}{c} s_{x}(t)\approx \frac{\partial m_{I}}{\partial x}|\Delta x|. \end{array}$$ (7)

Thus, the value of virion mutations is zero and are selectively neutral, while the value of infected cell mutations is maximized (Fig 2G and 2H).

In immunocompetent hosts, $\delta_{R}=\text{0}$ and thus the depletion of susceptible cells combined with the mobilized immune response clears the infection (Fig 2A-2C). However, in immunocompromised hosts, $\delta_{R}$ > 0, and the loss of IFN signalling and reversion to susceptibility of refractory cells creates a third phase of infection shortly after $t\;=\;\tau_{R}$. In this phase, there is a temporary abundance of susceptible cells as the first wave of refractory cells revert to susceptibility (Fig 2D-2F). The dynamics then settle down to a quasi-equilibrium (akin to the viral setpoint [46]) where infection size is limited by susceptible cell availability and the mobilized immune response (Fig 2D-2F; dark grey shaded region). Since $pB_{I}\to m_{V}m_{I}$ in the long term, the rebound in susceptible cells in immunocompromised hosts increases the value of virion mutations and tends to decrease the value of infected cell mutations (Fig 2G-2H). Moreover, the mobilization of the adaptive and humoral responses (Fig 2C and 2F) generates a direct effect on selection and increases the strength of selection for T cell and antibody evasion mutations from the second to third phase of infection (Fig 2G and 2H).

The time of onset of each phase is affected by the size of the viral inoculum, V(0). Smaller inoculums increase the amount of time required for the viral population to exhaust susceptible cells, thus prolonging the first phase of infection and delaying the onset of the second phase (Fig D in S1 Text). Although changes to the size of the viral inoculum affect the time of onset of each infection phase, they do not qualitatively change our key predictions, provided they are large enough to cause an infection (i.e., $V(\text{0})>{\text{1}\text{0}}^{-\text{4}}$ log(copies/mL; see Fig D in S1 Text and Jenner et al. [27]). Therefore, in what follows, we fix the initial inoculum at V(0)=4.5 log_10_(copies/mL) as in Jenner et al. [27]. For this inoculum size, the second and third phases of infection begin at approximately 4.5 and 9 days, respectively (Fig 2D and 2F).

### Immunodeficiencies speed and slow the evolution of individual mutations

Next, we ask how different immunodeficiencies affect the speed of viral evolution. The most straightforward prediction occurs for target immunodeficiencies, i.e., a production deficiency in the immunological variable directly targeted by the viral mutation. The direct effect of mutation x on viral fitness component $z\in \{m_{V},m_{I},f\}$ is an increasing function of the concentration of the target variable. Thus, target variable immunodeficiencies reduce the strength of selection on mutation x, slowing its evolution. This is intuitive; for example, if mutation x targets antibody evasion and antibody concentrations are reduced, there are fewer antibodies to “evade” and so mutation x will be less beneficial, weakening selection and, hence slowing the increase in frequency of mutation x. However, the interconnectedness of the immune response means that non-target immunodeficiencies may not only feedback on the target variable. Off-target effects directly impact unrelated mutations and trigger changes in mutational value by, e.g., affecting the availability of susceptible cells. In what follows, we consider these effects across the different phases of infection.

Our model predicts that mutations targeting infected cell evasion of neutrophils (decreased $\delta_{I,N}$) and evasion of macrophage (decreased $\delta_{V,M}$ and $\delta_{I,M}$) are under very weak selection, regardless of the size of the mutational effect (Fig B in S1 Text). Thus, we will ignore these three mutations and restrict our attention to mutations affecting cell entry (increased β), virion evasion of interferon, neutrophils, and antibodies (increased $\varepsilon_{F,I}$, or decreased $\delta_{V,N}$ and $\delta_{V,A}$, respectively), and infected cell evasion of T cells (decreased $\delta_{I,T}$).

#### Neutrophil and IFN deficiencies tend to speed viral evolution in the first phase of infection, and slow viral evolution in the second phase.

**Fig 3:**
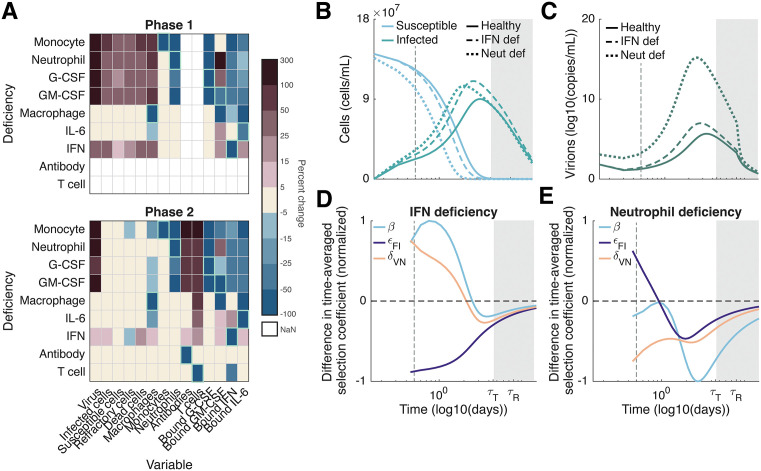
Viral evolution during acute infections is most strongly affected by neutrophil and IFN deficiencies. **A)** Percent change in each immunological variable (columns) resulting from an immunodeficiency in the indicated immune cell, cytokine, or antibody (rows) during acute infections (phase one and two; $\delta_{R}=\text{0}$). The percent change in each immunological variable is calculated as (X−Y)/X×100, where X and Y are the integrals of the variable over the length of the phase in immunodeficient and healthy hosts, respectively; the percent change in susceptible cells is calculated using the integral of $S_{max}-S(t)$. Green boxes indicate target variables. Note that NaN values reflect static T cell and antibody responses during the first phase of infection. **B-C)** Dynamics of susceptible and infected cells (**B**) and virions (**C**) during acute infection in healthy (solid lines), and immunocompromised hosts with IFN (dotted dashed lines) or neutrophil (dashed lines) deficiencies. **D-E)** Difference in time-averaged selection coefficient between IFN (**D**) and neutrophil (**E**) deficient and immunocompetent hosts, normalized by its maximum (absolute) value. During acute infections, IFN and neutrophil deficiencies tend to speed evolution of non-target mutations during the first phase of infection, before slowing evolution during the second phase of infection (see also Fig C i and viii in S1 Text). For acute infections, only mutations affecting cell entry, β, virion evasion of IFN, $\varepsilon_{F,I}$, and virion evasion of neutrophils, $\delta_{V,N}$, experience significant selection. **B-E)** White background: first phase of infection. Light grey background: second phase of infection. Dashed vertical grey line: 12 hours post-infection.

During the first two phases of infection, the innate response, consisting mainly of neutrophils, IFN, and macrophages, is largely responsible for infected cell and virion clearance (Fig 2B and 2E). Of the three, neutrophils are the most important for controlling the initial infection (Fig 3A). Neutrophils are recruited by G-CSF which is produced by monocytes generated through GM-CSF signalling (Fig 1). A deficiency in any component of this pathway either directly (i.e., a neutrophil deficiency) or indirectly (i.e., a G-CSF, GM-CSF, and/or monocyte deficiency) reduces neutrophil concentrations, sharply increasing infection size (Fig 3B and 3C). IFN deficiencies have a similar, but weaker effect. Since reduced neutrophil concentrations decrease the destruction of virions (decrease $m_{V}$), while reduced IFN concentrations lead to larger infection sizes (increase $B_{I}$), reductions in neutrophils and/or IFN increase the value of virion mutations. This increase tends to speed the evolution of non-target virion mutations targeting cell entry, neutrophil evasion, and IFN evasion during the first phase of infection (Figs 3D and 3E, see also Fig C i and viii in S1 Text).

Reduced concentrations of neutrophils and/or IFN and the increase in infection size during the first phase of infection lead to a more rapid depletion of susceptible cells, triggering an earlier onset of the second phase of infection. Since virion mutations are neutral during this phase (see equation (6)), the normalized time-averaged selection coefficient is larger in immunocompetent hosts by the end of the second phase of infection (Fig 3D and 3E). Thus, if refractory cells do not revert to susceptibility (i.e., there is no third phase of infection), mutations are at a higher frequency in immunocompetent hosts by the end of the infection.

#### T cell concentrations play a pivotal role in speeding or slowing viral evolution during phase three.

If refractory cells do not revert to susceptibility (i.e., $\delta_{R}$ = 0), the infection is cleared during phase two. Therefore, to provide a point of comparison for the immunodeficiency of interest, in this section we allow for the loss of refractory cells by setting $\delta_{R}>\text{0}$ in both immunodeficient and immunocompetent hosts.

**Fig 4:**
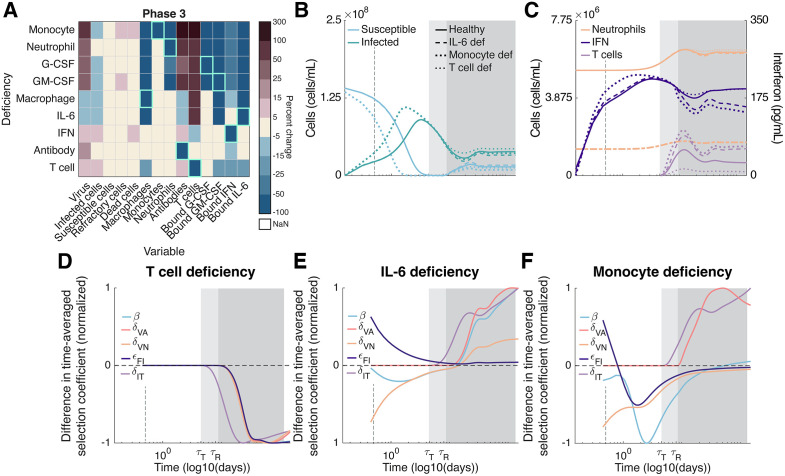
Immunodeficiencies affecting T cell concentrations strongly affect viral evolution during persistent infections. **A)** Percent change in each immunological variable (columns) resulting from an immunodeficiency in the indicated immune cell, cytokine, or antibody (rows) during persistent infections (i.e., $\delta_{R}$ > 0). The percent change in each immunological variable is calculated as (X−Y)/X×100, where X and Y are the integrals of the variable over the length of the phase in immunodeficient and healthy hosts, respectively; the percent change in susceptible cells is calculated using the integral of $S_{max}-S(t)$. Green boxes indicate target variables. Note that NaN values reflect static T cell and antibody responses during the first phase of infection. **B-C)** Dynamics of susceptible and infected cells **(B)**, and neutrophils, IFN, and T cells (**C**) during acute infection in healthy (solid lines), and immunocompromised hosts with IL-6 (dashed lines), monocyte (heavy dotted lines), and T cell (light dotted lines) deficiencies. **D-F)** Difference in time-averaged selection coefficient between T cell **(D)**, IL-6 **(E)**, and monocyte (**F**) deficient and immunocompetent hosts, normalized by its maximum (absolute) value. T cell killing of infected cells is a key determinant of the size of the infection and availability of susceptible cells during persistent infections. **D)** A deficiency in T cells will increase infection size and decrease the availability of susceptible cells, decreasing the value of virion mutations and so slowing the evolution of all mutations considered. **E)** A deficiency in IL-6 (either directly, or due to a macrophage deficiency; Fig C vi in S1 Text) will increase T cell concentrations and so speed the evolution of all mutations considered. **F)** A deficiency in neutrophils (either directly, or due to a deficiency in the cytokines G-CSF and GM-CSF or monocytes, as shown here; see also Fig C ii-iv in in S1 Text), will increase T cell concentrations, but will also reduce neutrophils and IFN. **B-F)** White background: first phase of infection. Light grey background: second phase of infection. Dark grey background: third phase of infection. Dashed vertical grey line: 12 hours post-infection.

During the third phase of infection, T cells play an important role in infected cell killing, thus reducing the infection size and increasing the availability of susceptible cells. Accordingly, the value of virion and infected cell mutations increases and decreases, respectively. T cell production is suppressed by bound IL-6 concentrations [48,49] generated through the stimulation of unbound IL-6 by neutrophils and monocytes, with unbound IL-6 generated by infected cells, monocytes, and macrophages (Fig 1; see also Section 1 in S1 Text). Consequently, while T cell deficiencies decrease T cell concentrations, deficiencies in neutrophils (arising either directly, or indirectly through deficiencies in G-CSF, GM-CSF, and/or monocytes), monocytes, macrophages and/or IL-6 will decrease bound IL-6 concentrations. Decreased bound IL-6 concentrations increase T cell concentrations (Fig 4A and 4C), decreasing infection size and increasing the availability of susceptible cells (Fig 4B). IFN and antibody deficiencies have limited effects on bound IL-6 or T cell concentrations (Fig 4A); this agrees with local sensitivity analysis results which show that only antibody concentrations are sensitive to small variations in antibody related parameters (Fig F in S1 Text).

Because a T cell deficiency both reduces the direct effect of T cell evasion and decreases the value of virion mutations by increasing susceptible cells, a T cell deficiency will slow the rate of increase of each of the mutations considered (Fig 4D). IL-6 and macrophage deficiencies have the opposite effect, as they increase T cell concentrations, accelerating the rate of increase in frequency of each of the five mutations considered (Fig 4E). As lower neutrophil concentrations increase infection size, T cell counts are also increased by lower neutrophil concentrations arising through deficiencies in monocytes, G-CSF, GM-CSF, and/or neutrophils, which also reduce IFN (Fig 4A). Thus, these deficiencies speed the evolution of mutations targeting cell entry, antibody evasion, and T cell evasion, but slow the evolution of mutations targeting IFN and neutrophil evasion by reducing the concentration of IFN and neutrophils (Fig 4F).

### Speed of evolution of multiple mutations

Finally, we ask how the speed at which individual mutations increase in frequency is affected by the presence of other segregating mutations. The possibility of genetic variation for multiple viral traits can yield fitness interactions between mutations (epistasis in fitness). Epistasis affects the speed of evolution by altering the fitness of a mutation depending on its genetic background and produces linkage disequilibrium (LD), i.e., non-random assortment, between mutations [50]. The amount of LD in deterministic models tends to scale with the strength of epistasis. For beneficial mutations, such as those studied here, these combined effects mean positive epistasis speeds evolution, whereas negative epistasis slows evolution [50–52] (S1 Text Section 4.5).

Under the same assumptions that allowed us to derive equation (6), we can calculate a weak selection approximation of epistasis between pairs of mutations (S1 Text Section 4.5). From this approximation, two predictions emerge. First, if both mutations directly affect the fitness of virions (i.e., $\delta_{V,N}$, $\delta_{V,A}$, $\varepsilon_{F,I}$, or β) or both mutations directly affect the fitness of infected cells, epistasis is positive, speeding their joint evolution (S1 Text Section 4.5). If instead, one mutation directly affects virion fitness, whereas the other mutation directly affects the fitness of infected cells (e.g., evasion of T cells), epistasis is negative, slowing their joint evolution (S1 Text Section 4.5).

**Fig 5:**
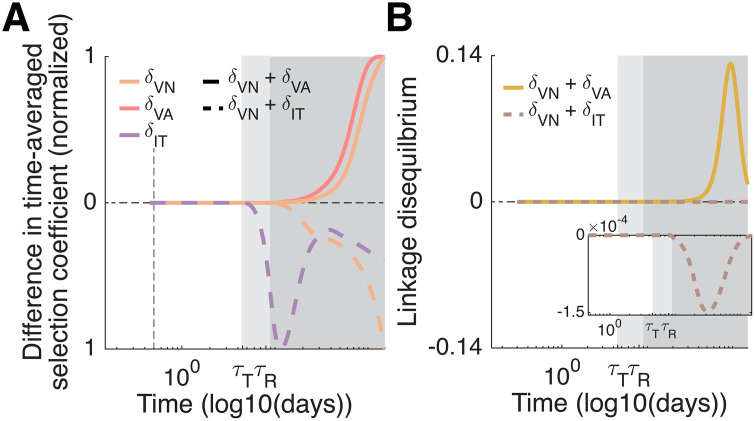
The sign of epistasis and the speed of viral evolution vary based upon the combination of mutations considered. **A)** Difference in time-averaged selection coefficients between immunocompromised and immunocompetent hosts during persistent infection with multiple mutations (i.e., $\delta_{VN}$+$\delta_{VA}$ and $\delta_{VN}$+$\delta_{IT}$) compared to persistent infection with single mutations (i.e., $\delta_{VN}$, $\delta_{VA}$, or $\delta_{IT}$ alone). The difference in the time-averaged selection coefficients is normalized by their respective maximum (absolute) value. **B)** Linkage disequilibrium between $\delta_{VN}$+$\delta_{VA}$ (solid yellow line) and $\delta_{VN}$+$\delta_{IT}$ (dashed brown line). **A-B)** When different mutations target immune evasion by the same life history stage (solid lines), i.e., infected cells or virions, the speed of evolution can be dramatically increased through positive epistasis and the production of significant amounts of linkage disequilibrium. Conversely, when one mutation targets T cell evasion, while the other targets virion evasion of the immune response (dashed lines) negative epistasis occurs. In contrast to positive epistasis, negative epistasis in our model is weak, and so is incapable of generating much linkage disequilibrium. That negative epistasis is weak arises due to the rapid turnover of virions relative to infected cells. White background: first phase of infection. Light grey background: second phase of infection. Dark grey background: third phase of infection.

Simulation results suggest that positive epistasis between virion mutations can dramatically speed evolution by enhancing fitness and by the generation of large amounts of LD, however the ability of negative epistasis between virion and infected cell mutations to slow evolution is weak (Fig 5). This occurs due to the source of epistasis, which depends upon the mutations under consideration. For example, mutations targeting cell entry and IFN evasion multiplicatively interact to affect the rate of infection of new cells, which will directly produce epistasis [53]. On the other hand, the source of negative epistasis in our model is strictly due to the division of the viral life cycle into virions and infected cells. This can be seen by supposing virion turnover is much more rapid than infected cell turnover, that is, $m_{V}\gg m_{I}$. As this difference grows, negative epistasis between virion and infected cell mutations becomes increasingly weak (S1 Text Section 4.5). Consequently, as our model parameterization assumes virion turnover is relatively rapid, negative epistasis is comparatively weak.

## Discussion

Acute infections can become persistent in immunodeficient hosts due to weakened immune defences. Such persistent infections can provide an ideal environment for the generation of, and selection for, viral mutations [36,54], increasing immune evasion [16,55,56] and viral replication [10,57,58]. Indeed, between-host models have shown that persistent infections can speed viral evolution across the host population [17–20,59]. However, this previous work largely assumed mutations are neutral within-host and thus ignores how immunodeficiencies alter the within-host environment and the implications for viral evolution. Here, we work towards filling this gap by studying viral evolution during primary infection in immunocompromised hosts using a mechanistic model of the immune response.

The viral mutations considered in our model can be classified as “virion” or “infected cell”, depending on the viral life stage they directly affect. The strength of selection for virion mutations tends to be positively correlated with the availability of susceptible cells and so follows a U-shaped dynamic through the phases of infection (Fig 2G and 2H). Virion mutations tend to be under strong selection at the outset of the infection when susceptible cells are abundant (phase one), selectively neutral once susceptible cells have been exhausted (phase two), and rebound in value as refractory cells revert to susceptibility in persistent infections (phase three). Conversely, the strength of selection on infected cell mutations tends to be negatively correlated with the availability of susceptible cells and so follows an inverted U-shaped dynamic through the phases of infection.

The strength of selection on virion and infected cell mutations also depends on the type and severity of immunodeficiency. During an acute infection (phases one and two), innate immune immunodeficiencies, specifically deficiencies in IFN, neutrophils, monocytes and/or the cytokines G-CSF and GM-CSF, play the most significant evolutionary role. Although such deficiencies strengthen selection on non-target virion mutations during the first phase of infection, selection is weakened during the second phase. Thus, by the end of an acute infection, the frequency of virion mutations tends to be lower in hosts with these immunodeficiencies (Fig 3D and 3E). During a persistent infection (phase three), T cell deficiencies increase infection size and so decrease the availability of susceptible cells. This slows the evolution of virion mutations, while also reducing selection on T cell evasion (Fig 4D). Conversely, an IL-6 and/or macrophage deficiency overstimulates T cells, thus speeding the evolution of virion mutations and T cell evasion (Fig 4E). While reduced neutrophil concentrations increase T cell counts, they also reduce IFN concentrations and so speed the evolution of mutations targeting cell entry, and antibody and T cell evasion, but slow the evolution of evasion of neutrophil and IFN (Fig 4F). These predictions align with clinical case reports. For example, Khatamzas et al. [60] described a persistent SARS-CoV-2 infection in a patient with B cell lymphoma who had a strong CD8 + T cell response to viral proteins, despite receiving B cell depleting treatment. In this individual, the dominant SARS-CoV-2 mutations altered CD8 + T cell epitopes, presumably in response to the selective pressure exerted by CD8 + T cells. Conversely, Sonnleitner et al. [61] described a persistent SARS-CoV-2 infection in a patient with stage IVa small cell lymphocytic lymphoma who displayed a weakened humoral response and complete lack of functional CD8 + T cell response. Longitudinal viral sequencing revealed a high degree of within-host evolution, with mutations predominantly found in the SARS-CoV-2 spike protein targeted by antibodies, suggesting reduced CD8 + T cell selection pressure.

Previous work using between-host models indicates that fitness valleys (i.e., negative epistasis), are necessary for immunocompromised hosts to promote the emergence of novel viral strains [19,20]. While fitness valleys are important at the between-host level, at the within-host level, it is probable that positive epistasis is more likely to produce genetically distinct strains by accelerating evolution during persistent infections where viral diversity is more common. Indeed, our model indicates positive epistasis occurs between combinations of virion mutations and can significantly accelerate within-host evolution (Fig 5). Conversely, negative epistasis, which occurs between virion mutations and mutations affecting T cell evasion, has a negligible effect (Fig 5). Importantly, epistasis emerges in our model due to the immunological dynamics (i.e., from the phenotype-fitness mapping); previous work generated epistasis by building it into the genotype-phenotype map [19–21].

Macrophages are an important component of the innate immune response, helping to bridge the innate and adaptive immune response [62] and clear cellular debris [63]. As macrophages are primarily an orchestrator of the immune response, our model indicates evasion of macrophages is under weak selection (Fig B in S1 Text) and macrophage deficiencies have limited evolutionary impact during acute infection. However, during persistent infections, macrophage deficiencies overstimulate T cells, speeding viral evolution. Mathematical modelling indicates macrophage deficiencies through dysregulated monocyte-to-macrophage differentiation, are predictive of disease severity [27], as they cause T cell lymphopenia and hyperinflammation. Clinical studies have similarly reported shifts in the proportions of monocyte and macrophage subsets in the blood and lungs of severe COVID-19 patients, causing reductions to T cell recruitment of T cell and counts [64]. In combination with our analysis, this would suggest severe disease can also provide an optimal environment for within-host evolution.

To isolate how immunodeficiencies affect the orchestration of the immune response and the condition of the immunocompromised host at homeostasis, we intentionally constrained each immunodeficiency to reducing the production of a single immunological state variable. Real immunodeficiencies, however, involve multiple aspects of the immune response. For example, hosts with uncontrolled HIV show decreased production of naïve T cells, and CD4 + T cell lymphopenia [65], resulting in a failure to produce antibodies [66]. Importantly, most clinically observed immunodeficiencies exhibit decreased T cells (see S1 Text Section 2.1), which our analysis reveals will slow viral evolution (Fig 4D). A notable exception are hosts with B cell deficiencies and/or lymphomas [67], who have weakened antibody responses and neutrophil deficiencies [37,68], the latter of which speeds the evolution of T cell evasion (Fig 4F).

In a secondary infection, the adaptive and humoral response will more rapidly activate leading to an earlier onset of selection for antibody and T cell evasion. Although beyond the scope of the current work, this raises the question of whether these mutations should be more strongly selected for in a primary infection in an immunocompromised host, or in a secondary infection in an immunocompetent host? Owing to the heightened value of virion mutations at the beginning of the infection when susceptible cells are abundant, our analysis suggests that antibody evasion is more likely to be selected for in a secondary infection in an immunocompetent host. Conversely, as the value of infected cell mutations tends to be inversely correlated with the availability of susceptible cells, T cell mutations are more likely to experience stronger selection in a primary infection in an immunocompromised host.

To isolate the role of selection, we ignored stochasticity. Stochasticity is expected to have two effects. First, genetic drift can lead to the chance loss of viral mutations at small population sizes. Because we assumed mutations were present at low frequencies in the viral inoculum, genetic drift would be most likely to play a significant role during the establishment of the infection. During this initial stage, the abundance of susceptible cells means that immunodeficiencies play a limited role, and so we would expect genetic drift to play a similar role in both immunocompetent and immunodeficient individuals. Second, as we assumed mutations were present at low frequencies in the viral inoculum, we ignored the (stochastic) generation of *de novo* mutations. It is well understood that, owing to the exponential within-host growth of viral populations during acute infections, most *de novo* mutations in acute infections will appear just before the susceptible cells are exhausted [69,70], greatly restricting the amount of time for selection to operate. This constraint is alleviated in persistent infections, providing another reason for more rapid adaptation in immunocompromised hosts, as compared to chains of acute infections [70]. We also assumed each mutation had a single beneficial effect on viral fitness and so ignored potential costs or trade-offs. Fitness trade-offs, however, are likely to affect the forward transmission process as within-host evolution is unlikely to optimize transmission between-hosts. Indeed, there are some data in SARS-CoV-2 evolution in immunocompromised hosts of a trade-off between antibody evasion and between-host transmissibility [58]. Thus, integrating our within-host evolutionary analysis into a between-host nested framework [71] represents an important next step for future work to quantify the population-level risks posed by within-host evolution during persistent infections.

## Supporting information

S1 Text**Fig A. Changes in the immunological response in hosts with varying degrees of immunodeficiencies. i-iii)** Percent change in each immunological variable (columns) resulting from an immunodeficiency in a key immune cell, cytokine, or antibody (rows) during acute infections (phase one and two; $\delta_{R}=\text{0}$). The percent change in each immunological variable is calculated as (X−Y)/X×100, where X and Y are the integrals of the indicated variable over the length of the phase in immunodeficient and healthy hosts, respectively, while the percent change in susceptible cells is calculated using the integral of $S_{max}-S(t)$. Note that NaN values reflect static T cell and antibody responses during the first phase of infection. **i)** Severity of immunodeficiencies reduced by 25% from its baseline value (shown in **ii**). **ii)** Severity of immunodeficiencies used in the main text (baseline value). **iii)** Severity of immunodeficiencies increased by 25% from its baseline value (shown in **ii**). Increasing the severity of the immunodeficiencies results in a quantitative, but not qualitative, change in the concentrations of the different immunological variables. **Fig B. Comparison of different approximations of the selection coefficient.** Selection coefficients numerically calculated four ways: (1) using formula (S36), with $r_{x_{m}}$ and $r_{x_{w}}$ calculated using the matrix in equation (S40) (dashed-dot lines); (2) using formula (S36), with $r_{x_{m}}$ and $r_{x_{w}}$ given by equation (S44) (dotted lines); (3) applying equation (S45) to simulation data (solid lines); or (4) applying equation (S45) to simulation data, except by measuring frequency amongst virions (dashed lines) for **i)** β, **ii)** $\varepsilon_{FI}$, **iii)** $\delta_{VN}$, **iv)** $\delta_{VA}$, **v)** $\delta_{IT}$, **vi)** $\delta_{VM}$, **vii)** $\delta_{IM}$, and **viii)** $\delta_{IN}$ mutations. Virion and infected cell evasion of macrophage (**vi** and **vii**), and infected cell evasion of neutrophils (**viii**) are under very weak selection, even though the mutational effect size is maximal. **i-viii)** Dashed vertical grey line: 12 hours post-infection. White background: first phase of infection. Light grey background: second phase of infection. Dark grey background: third phase of infection. **Fig C. Qualitative similarities in evolutionary dynamics are determined by the type of immunodeficiency.** Time-averaged selection coefficient calculated using formula (S36), with $r_{x_{m}}$ and $r_{x_{w}}$ calculated as the dominant eigenvalue of matrix (Eq. S40) (dashed lines), or by applying equation (Eq. S45) to simulation data (solid lines). Qualitatively similar viral evolutionary dynamics emerge for **i)** neutrophil, **ii)** monocyte, **iii)** G-CSF, and **iv)** GM-CSF deficiencies (orange background), **v)** macrophage and **vi)** IL-6 deficiencies (red background), while **vii)** T cell (purple background) and **viii)** IFN (blue background) deficiencies show distinct dynamics. The divergence between these measures at the beginning of the infection arises due to two sources: (1) estimating the selection coefficient from the simulation data only takes into account the frequency of mutations in infected cells, whereas the true selection coefficient is a weighted average of infected cells and virions, and (2) at the beginning of the infection, there are only virions present and there is a delay owing to the eclipse phase before infected cells appear. **i-viii)** Dashed vertical grey line: 12 hours post-infection. White background: first phase of infection. Light grey background: second phase of infection. Dark grey background: third phase of infection. **Fig D. Inoculum size influences the onset of infection phases.** Dynamics of **i)** susceptible and refractory cells, **ii)** infected cells and virions, **iii)** macrophages and neutrophils, **iv)** interferon, **v)** T cells, and **vi)** antibodies for initial viral inoculum size of 1, 4.5, and 9 log_10_(copies/mL). Larger inoculum sizes shorten (shift left) the first phase of infection due to higher viral loads and faster depletion of susceptible cells, while the reverse is true for smaller inoculum sizes. **i-vi)** White background: first phase of infection; light grey background: second phase of infection; dark grey background: third phase of infection regions for an inoculum of 4.5 log_10_(copies/mL), as in the results shown in the main text. **Fig E. Model prediction compared to viral loads from patients hospitalized during the first and second waves in Germany, Singapore, and Canada**. Data (open grey circles) from patients hospitalized during the first and second waves used for model calibration in Jenner et al. (Singapore and Germany; original data from Goyal et al. [36]) and Gazeau et al. [33] (Canada; original data from Rébillard et al. [37]) compared to average model predicted viral load (Eq. 1 in revised Main Text). Dashed horizontal line: level of detection [33]. DSO: days from symptom onset. **Fig F. Antibody parameter local sensitivity results.** Percent change in each immunological variable (columns) resulting from **i)** a 20% decrease or **ii)** a 20% increase in antibody parameter value (rows). The percent change in each immunological variable is calculated as (X−Y)/X×100, where X and Y are the maximum value of the indicated variable in healthy and immunocompromised hosts, respectively, during the third phase of the infection; the percent change in susceptible cells is calculated with respect to the difference $S_{max}-S(t)$. Percent changes of less than 20% are considered to be insensitive to parameter value changes. **Table A. Parameter values for the immunological model.** Parameters added in the present study are identified by shaded grey backgrounds.(PDF)
