# Supplementary material for: Viral evolution during primary infection in immunocompromised hosts
Source: PLoS Comput Biol. 2026 Feb 25;22(2):e1013967. doi: 10.1371/journal.pcbi.1013967 (PMC12935273; doi:10.1371/journal.pcbi.1013967)
Supplement: S1 Text — Fig A. Changes in the immunological response in hosts with varying degrees of immunodeficiencies. i-iii) Percent change in each immunological variable (columns) resulting from an immunodeficiency in a key immune cell, cytokine, or antibody (rows) during acute infections (phase one and two; δR=0). The percent change in each immunological variable is calculated as (X−Y)/X×100, where X and Y are the integrals of the indicated variable over the length of the phase in immunodeficient and healthy hosts, respectively, while the percent change in susceptible cells is calculated using the integral of Smax−S(t). Note that NaN values reflect static T cell and antibody responses during the first phase of infection. i) Severity of immunodeficiencies reduced by 25% from its baseline value (shown in ii). ii) Severity of immunodeficiencies used in the main text (baseline value). iii) Severity of immunodeficiencies increased by 25% from its baseline value (shown in ii). Increasing the severity of the immunodeficiencies results in a quantitative, but not qualitative, change in the concentrations of the different immunological variables. Fig B. Comparison of different approximations of the selection coefficient. Selection coefficients numerically calculated four ways: (1) using formula (S36), with rxm and rxw calculated using the matrix in equation (S40) (dashed-dot lines); (2) using formula (S36), with rxm and rxw given by equation (S44) (dotted lines); (3) applying equation (S45) to simulation data (solid lines); or (4) applying equation (S45) to simulation data, except by measuring frequency amongst virions (dashed lines) for i) β, ii) εFI, iii) δVN, iv) δVA, v) δIT, vi) δVM, vii) δIM, and viii) δIN mutations. Virion and infected cell evasion of macrophage (vi and vii), and infected cell evasion of neutrophils (viii) are under very weak selection, even though the mutational effect size is maximal. i-viii) Dashed vertical grey line: 12 hours post-infection. White background: first p [file pcbi.1013967.s001.pdf]

Supplementary Information to: Viral evolution during primary infection in immunocompromised hosts

Morgan Craig, Xiaoyan Deng, and David V. McLeod

Correspondence to: morgan.craig@umontreal.ca, david.mcleod@umontreal.ca

1. Immunological model of primary infection

Virions, $V(t)$, infect susceptible cells, $S(t)$, producing infected cells, $I(t)$, via mass action with rate constant β. Infected cells initially enter an eclipse phase lasting τ_I days during which they are non-productively infected. Following the eclipse phase, lysis occurs with a burst size of p virions.

Virions naturally decay at a per-capita rate d_V and are destroyed by neutrophils, $N(t)$, inflammatory macrophages, $M_{\Phi I}(t)$, and antibodies, $A(t)$. Both neutrophils and inflammatory macrophages remove virions through mass action interactions given by $\delta_{V,N}NI$ and $\delta_{V,M}M_{\Phi,I}I$, where $\delta_{V,N}$ and $\delta_{V,M}$ are the per-capita killing rates of neutrophils and macrophages, respectively. Antibodies neutralize virions via Hill function kinetics given by

$$\delta_{V,A}\frac{A^{h_A}}{\epsilon_{V,A}^{h_A}+A^{h_A}},$$

where $\delta_{V,A}$ is the maximal per capita rate of neutralization, $\epsilon_{V,A}$ is the antibody concentration corresponding to $0.5\delta_{V,A}$ (i.e., half-effect concentration), and h_A is the Hill coefficient.

Infected cells die at a per-capita rate d_I, and are killed by neutrophils, inflammatory macrophages, and T cells, $T(t)$. Macrophages kill infected cells through mass action kinetics at per-capita rate $\delta_{I,M}$, whereas neutrophils and T cells kill infected cells at saturable rates given by

$$\frac{\delta_{I,N}N^{h_N}}{N^{h_N}+IC_{50,N}^{h_N}}$$

and

$$\frac{\delta_{I,T}T}{\epsilon_{\delta,T}+I^{h_T}},$$

where $\delta_{I,N}$ and $\delta_{I,T}$ are the maximal killing rates of neutrophils and T cells, respectively, h_N and h_T are Hill coefficients, $IC_{50,N}$ is the half-effect neutrophil concentration, and $\epsilon_{\delta,T}$ denotes the half-effect concentration of T cells. This form of T cell killing implies that infected cell clearance by T

cells is maximal when infected cell concentrations are low. Infected cells can become refractory to infection, $R(t)$, through signalling by Type-I interferon (IFN), $F_b(t)$:

$$\frac{\beta F_b}{F_b + \epsilon_{F,I}},$$

where $\epsilon_{F,I}$ is the half-effect concentration. After τ_R time units, refractory cells revert to susceptibility at a per-capita rate δ_R. The delay[1] in reversion accounts for the loss of increased Type I IFN signalling and IFN stimulated genes[2] through e.g., receptor internalization and downregulation[3].

Susceptible and refractory cells are killed by neutrophil toxicity through bystander damage at a maximal rate of neutrophil inflicted damage of δ_N according to:

$$\frac{\rho \delta_N (S + R) N^{h_N}}{N^{h_N} + IC_{50,N}^{h_N}},$$

where ρ modulates the extent of bystander cell death. These cells also undergo logistic growth, which depends on the concentration of susceptible, refractory, and infected cells, as well as the concentration of dead (and damaged) cells, $D(t)$. Dead cells are generated by death or damage of infected, susceptible, and refractory cells. Dead cells naturally decay and are destroyed by macrophages.

Let

$$m_V \equiv d_V + \delta_{V,N} N + \delta_{V,M} M_{\Phi I} + \delta_{V,A} \frac{A^{h_A}}{\epsilon_{V,A}^{h_A} + A^{h_A}} \tag{S1}$$

$$m_I \equiv d_I + \frac{\delta_{I,N} N^{h_N}}{N^{h_N} + IC_{50,N}^{h_N}} + \delta_{I,M} M_{\Phi \mathrm{I}} + \frac{\delta_{I,T} T}{\epsilon_{\delta,T} + I^{h_T}} \tag{S2}$$

denote the per-capita decay rates of virions and infected cells, respectively. Then the dynamics of virions, and infected, susceptible, refractory, and dead cells are given by the following equations:

$$\frac{dV}{dt} = pI - m_V V, \tag{S3}$$

$$\frac{dI}{dt} = \frac{\beta \epsilon_{F,I}}{\epsilon_{F,I} + F_b} S(t - \tau_I) V(t - \tau_I) - m_I I, \tag{S4}$$

$$\frac{dS}{dt} = \kappa_S \left(1 - \frac{C}{S_{max}}\right) S - \left(\beta V + \frac{\rho \delta_N N^{h_N}}{N^{h_N} + IC_{50,N}^{h_N}}\right) S + \delta_R R(t - \tau_R), \tag{S5}$$

$$\frac{dR}{dt} = \kappa_S \left(1 - \frac{C}{S_{max}}\right) R + \frac{\beta F_b}{F_b + \epsilon_{F,I}} S(t - \tau_I) V(t - \tau_I) - \left(\frac{\rho \delta_N N^{h_N}}{N^{h_N} + IC_{50,N}^{h_N}}\right) R - \delta_R R(t - \tau_R), \tag{S6}$$

$$\frac{dD}{dt} = m_I I + \frac{\rho \delta_N (S + R) N^{h_N}}{N^{h_N} + IC_{50,N}^{h_N}} - (d_D + (\delta_{D,M_\Phi} - \delta_{M_\Phi,D})(M_{\Phi R} + M_{\Phi I}))D, \tag{S7}$$

where $C \equiv S + I + R + D$ is the total density of cells, regardless of state.

Infection first induces an innate response by neutrophils, $N(t)$, and inflammatory macrophages, $M_{\Phi I}(t)$. Neutrophils are generated by the bound cytokines interleukin-6 (IL-6; $L_b(t)$) and granulocyte colony-stimulating factor (G-CSF; $C_b(t)$) at rate

$$\left(N_{prod}^* + \left(\psi_N^{max} - N_{prod}^*\right)\frac{C_b/(\zeta_C N) - C_{bF}^*}{C_b/(\zeta_C N) - C_{bF}^* + \epsilon_{C,N}}\right),$$

which is proportional to the concentration of neutrophils in the bone marrow, N_R^*, which is taken to be a constant for simplicity. Here, N_{prod}^* is homeostatic rate of release of neutrophils from the bone marrow, ψ_N^{max} is the maximal reservoir release rate, C_b is the bound concentration of G-CSF (here and in what follows we use a subscript b and u to denote bound and unbound cytokines, respectively), ζ_C is a conversion factor (see below), C_{bF}^* denotes the fraction of bound neutrophil receptors at homeostasis, and $\epsilon_{C,N}$ is the half-effect parameter. Neutrophils have a natural per-capita death rate of d_N. Neutrophil dynamics are given by

$$\frac{dN}{dt} = \left(N_{prod}^* + \left(\psi_N^{max} - N_{prod}^*\right)\frac{C_b/(\zeta_C N) - C_{bF}^*}{C_b/(\zeta_C N) - C_{bF}^* + \epsilon_{C,N}}\right)N_R^* + \frac{p_{N,L}L_b}{L_b + \epsilon_{L,N}} - d_N N. \quad \text{(S8)}$$

Inflammatory macrophages are differentiated from monocytes, $M(t)$, at a per-capita rate that depends on the cytokines IL-6 and granulocyte macrophage-colony stimulating factor (GM-CSF; $G_b(t)$) given by

$$\theta_M \equiv \frac{p_{M_{\Phi I,G}} G_b^{h_{M,M\Phi}}}{G_b^{h_{M,M\Phi}} + \epsilon_{G,M_{\Phi I}}^{h_{M,M\Phi}}} + \frac{p_{M_{\Phi I,L}} L_b}{L_b + \epsilon_{L,M_\Phi}},$$

where $p_{M_{\Phi I,G}}$ is the maximal rate of inflammatory macrophage production, $h_{M,M\Phi}$ is the Hill coefficient, and $\epsilon_{G,M\Phi}$ is the half-effect concentration. Inflammatory macrophages are also converted from tissue-resident macrophages, $M_{\Phi R}(t)$, at a per-capita rate $a_{I,M\Phi}$ depending on infected and dead cells:

$$\theta_{M_{\Phi R,I}} \equiv a_{I,M\Phi}(I + D).$$

In turn, inflammatory macrophages convert to tissue-resident macrophages at a per-capita rate κ_{M_Φ} depending on tissue-resident macrophages and virions:

$$\theta_{M_{\Phi I,R}} \equiv \left(1 - \frac{M_{\Phi R}}{M_{\Phi max}}\right)\frac{\kappa_{M_\Phi}}{V + \epsilon_{V,M_\Phi}},$$

where ϵ_{V,M_Φ} is the half-effect parameter. Macrophages are destroyed through phagocytosis and natural decay at per-capita rates $d_{M_{\Phi I}}$ and $d_{M_{\Phi R}}$. Inflammatory and tissue-resident macrophage dynamics are given by

$$\frac{dM_{\Phi I}}{dt} = \theta_{M_{\Phi R,I}} M_{\Phi R} + \theta_M M - \left(d_{M_{\Phi I}} + \delta_{M_\Phi,D} D + \theta_{M_{\Phi I,R}}\right)M_{\Phi I}, \quad \text{(S9)}$$

$$\frac{dM_{\Phi R}}{dt} = \theta_{M_{\Phi I,R}} M_{\Phi I} - \left(d_{M_{\Phi R}} + \delta_{M_\Phi,D} D + \theta_{M_{\Phi R,I}}\right)M_{\Phi R}. \quad \text{(S10)}$$

Similar to neutrophils, monocytes are generated by the cytokine GM-CSF at a rate proportional to the concentration of monocyte precursors in the bone marrow, M_R^* (taken to be a constant for simplicity):

$$\left(M_{prod}^* + \left(\psi_M^{max} - M_{prod}^*\right)\frac{G_b^{h_M}}{G_b^{h_M} + \epsilon_{G,M}^{h_M}}\right)M_R^*,$$

where M_{prod}^* is the marrow reservoir concentration of monocytes at homeostasis, ψ_M^{max} is maximal reservoir release rate, h_M is the Hill coefficient, and $\epsilon_{G,M}$ is the half-effect concentration. Monocytes are also generated nonlinearly through stimulation by infected cells at a maximal rate of $p_{M,I}$ with half-effect concentration $\epsilon_{I,M}$. Monocyte dynamics are given by

$$\frac{dM}{dt} = \left(M_{prod}^* + \left(\psi_M^{max} - M_{prod}^*\right)\frac{G_b^{h_M}}{G_b^{h_M} + \epsilon_{G,M}^{h_M}}\right)M_R^* + \frac{p_{M,I}I}{I + \epsilon_{I,M}}M - (d_M + \theta_M)M. \quad \text{(S11)}$$

While the innate response is being mobilized, the humoral response mediated by antibodies, $A(t)$, is generated by the presence of virions. The production of antibody by virions starts after τ_A days. The delay in antibody production accounts for the time required for the uptake of antigen by antigen presenting cells and their presentation to CD4+ T cells, the initial production of antibodies by short-lived plasma cells after CD4+ T cell priming, and the production of long-lived plasma cells and memory B cells from germinal centre B cells undergoing somatic hypermutation[4]. Antibodies neutralize virions and are lost due to natural decay at per-capita rate d_A. Their dynamics are given by

$$\frac{dA}{dt} = p_{A,V}V(t - \tau_A) - d_A A - \delta_{V,A}\frac{A^{h_A}V}{\epsilon_{V,A}^{h_A} + A^{h_A}}. \quad \text{(S12)}$$

After τ_T days, the adaptive immune response mediated by CD8+ effector T cells, $T(t)$, is activated. The activation delay accounts for the time necessary for naïve CD8+ T cells to be presented antigen, and to convert to the effector phenotype following a primary exposure[5-8]. CD8+ T cells are recruited by infected cells through a process mediated by IL-6[9] at maximal rate $p_{T,I}$ and half-effect concentration $\epsilon_{L,T}$, are stimulated by IFN at a maximal rate of $p_{T,F}$ with half-effect concentration $\epsilon_{F,T}$, and are lost due to natural death at per-capita rate d_T. Their dynamics are given by

$$\frac{dT}{dt} = \frac{p_{T,I}\epsilon_{L,T}}{L_b + \epsilon_{L,T}}I(t - \tau_T) + \frac{p_{T,F}F_b}{F_b + \epsilon_{F,T}}T - d_T T. \quad \text{(S13)}$$

The action of the immune system is mediated by cytokines. We model unbound and bound cytokines, with bound cytokines responsible for immune effects. Let $Y_u(t)$ and $Y_b(t)$ denote the concentration of unbound and bound cytokine Y at time t, and $Y_{prod}(t)$ denote the rate of endogenous cytokine production. Then the general pharmacokinetic model of cytokine unbinding/binding is given by

$$\frac{dY_u}{dt} = Y_{prod} - k_{lin}Y_u - k_b(X\zeta - Y_b)(Y_u)^{POW} + k_u Y_b,$$
$$\frac{dY_b}{dt} = -k_{in}Y_b + k_b(X\zeta - Y_b)(Y_u)^{POW} - k_u Y_b.$$

Here, k_b and k_u are the binding and unbinding rates, respectively, k_{in} is the rate of bound cytokine internalization, k_{lin} is the rate of elimination, POW is a stoichiometric constant, X is the sum of all cells modulated by the cytokine, and ζ is a scaling factor satisfying

$$\mathrm{X}\zeta = \hat{p} Y_{MW} \mathrm{K} 10^n \mathrm{X},$$

in concentration units of pg/mL. We used the equation $Y_{MW} = MM/N_A$ to calculate the molecular weight of each cytokine, where MM is the molar mass and $N_A = 6.02214 \times 10^{23}$ is Avogadro's number. In the equation above, $\hat{p}$ is a constant relating the stoichiometry between cytokine molecules and their receptors, K is the number of receptors specific to each cytokine on a cell's surface, and 10^n is a factor correcting for cellular units, giving:

$$\zeta_F = \frac{MM_F}{N_A}\left(K_{F,T} + K_{F,I}\right) \cdot \left(\frac{10^{-3}}{5000}\right),$$
$$\zeta_L = \frac{MM_L}{N_A}\left(K_{L,N} + K_{L,T} + K_{L,M}\right) \cdot \left(\frac{10^{-3}}{5000}\right),$$
$$\zeta_G = \frac{MM_G}{N_A} K_{G,M} \cdot \left(\frac{10^{-3}}{5000}\right),$$
$$\zeta_C = \hat{p}\frac{MM_C}{N_A} K_{C,N} \cdot \left(\frac{10^{1}}{5000}\right).$$

In our immunological model of primary infection, there are four key cytokines[5]: Type-I interferon (IFN), interleukin-6 (IL-6), granulocyte colony-stimulating factor (G-CSF), and granulocyte macrophage-colony stimulating factor (GM-CSF).

Let $p_{A,B}$ and $\eta_{A,B}$ be the maximal production rate and half-effect concentration, respectively, of cytokine A stimulated by cell B. Unbound IFN, $F_u(t)$, is produced by infected cells, monocytes, and inflammatory macrophages. IFN binds to infected cells and CD8+ T cells, producing bound IFN, $F_b(t)$, that leads to refractory cells. The dynamics of IFN are captured by

$$\frac{dF_u}{dt} = \frac{p_{F,I} I}{I + \eta_{F,I}} + \frac{p_{F,M} M}{M + \eta_{F,M}} + \frac{p_{F,M_{\Phi I}} M_{\Phi I}}{M_{\Phi I} + \eta_{F,M_{\Phi I}}} - k_{lin_F} F_u - k_{b_F}\big((I+T)\zeta_F - F_b\big)F_u + k_{u_F} F_b, \tag{S14}$$

$$\frac{dF_b}{dt} = -(k_{in_F} + k_{u_F})F_b + k_{b_F}\big((I+T)\zeta_F - F_b\big)F_u. \tag{S15}$$

Unbound IL-6, $L_u(t)$, is produced by infected cells, monocytes, and inflammatory macrophages. IL-6 binds to neutrophils, monocytes, and CD8+ T cells, forming bound IL-6, $L_b(t)$, which stimulates neutrophil production and the differentiation of monocytes into inflammatory macrophage, and inhibits the production of CD8+ T cells. The dynamics of IL-6 are given by

$$\frac{dL_u}{dt} = \frac{p_{L,I} I}{I + \eta_{L,I}} + \frac{p_{L,M} M}{M + \eta_{L,M}} + \frac{p_{L,M_{\Phi I}} M_{\Phi I}}{M_{\Phi I} + \eta_{L,M_{\Phi \mathrm{I}}}} - k_{lin_L} L_u - k_{b_L}\big((N+M+T)\zeta_L - L_b\big)L_u + k_{u_L} L_b, \tag{S16}$$

$$\frac{dL_b}{dt} = -\left(k_{in_L} + k_{u_L}\right)L_b + k_{b_L}\big((N+M+T)\zeta_L - L_b\big)L_u. \tag{S17}$$

Unbound G-CSF, $C_u(t)$, is produced by monocytes. G-CSF binds to neutrophils, forming bound G-CSF, $C_b(t)$, which stimulates neutrophil production. The dynamics of G-CSF are given by

$$\frac{dC_u}{dt} = \frac{p_{C,M}M}{M + \eta_{C,M}} - k_{lin_C}C_u - k_{b_C}(N\zeta_C - C_b)(C_u)^{POW} + k_{u_C}C_b, \tag{S18}$$

$$\frac{dC_b}{dt} = -k_{in_C}C_b + k_{b_C}(N\zeta_C - C_b)(C_u)^{POW} - k_{u_C}C_b. \tag{S19}$$

Finally, unbound GM-CSF, $G_u(t)$, is produced by monocytes and inflammatory macrophages and binds to monocytes, forming bound GM-CSF, $G_b(t)$. Bound GM-CSF produces monocytes and induces differentiation of monocytes into inflammatory macrophages. The dynamics of GM-CSF are given by

$$\frac{dG_u}{dt} = \frac{p_{G,M}M}{M + \eta_{G,M}} + \frac{p_{G,M_{\Phi I}}M_{\Phi I}}{M_{\Phi I} + \eta_{G,M_\Phi}} - k_{lin_G}G_u - k_{b_G}(M\zeta_G - G_b)G_u + k_{u_G}G_b, \tag{S20}$$

$$\frac{dG_b}{dt} = -k_{in_G}G_b + k_{b_G}(M\zeta_G - G_b)G_u - k_u G_b. \tag{S21}$$

2. Immunological dynamics in immunocompromised hosts

Next, we modify the immunological model to allow for immunodeficiencies. We assume immunodeficiencies have two effects. First, they lead to prolonged infection due to the loss of IFN effects on refractory cells. To capture this, after τ_R time units, refractory cells revert to susceptibility at a per-capita rate δ_R in immunocompromised hosts (in immunocompetent hosts, $\delta_R = 0$). Second, immunodeficiencies reduce the rate of production, and hence availability, of different aspects of the immune response. For example, a monocyte production deficiency translates to a lower concentration of monocytes at homeostasis and during infection.

Let λ_i denote a production deficiency in immune variable $i \in \{N, M_{\Phi,I}, A, T, F_u, M, L_u, C_u, G_u\}$. Then production deficiencies modify the model as follows:

$$\frac{dN}{dt} = \lambda_N \left[\left(N^*_{prod} + \left(\psi_N^{max} - N^*_{prod}\right) \frac{C_b/(A_C N) - C^*_{bF}}{C_b/(A_C N) - C^*_{bF} + \epsilon_{C,N}} \right) N^*_R + \frac{p_{N,L}L_b}{L_b + \epsilon_{L,N}} \right] - d_N N, \tag{S24}$$

$$\frac{dM_{\Phi I}}{dt} = \theta_{M_{\Phi R,I}} M_{\Phi R} + \lambda_{M_{\Phi R}} \theta_M M - \left(d_{M_{\Phi I}} + \delta_{M_\Phi,D} D + \theta_{M_{\Phi I,R}}\right) M_{\Phi I}, \tag{S25}$$

$$\frac{dA}{dt} = \lambda_A p_{A,V} V - d_A A - \delta_{V,A} \frac{A^{h_A} V}{\epsilon_{V,A}^{h_A} + A^{h_A}}, \tag{S26}$$

$$\frac{dT}{dt} = \lambda_T \left[\frac{p_{T,I}\epsilon_{L,T}}{L_b + \epsilon_{L,T}} I(t - \tau_T) + \frac{p_{T,F}F_b}{F_b + \epsilon_{F,T}} T \right] - d_T T, \tag{S27}$$

$$\frac{dF_u}{dt} = \lambda_F \left[\frac{p_{F,I}I}{I + \eta_{F,I}} + \frac{p_{F,M}M}{M + \eta_{F,M}} + \frac{p_{F,M_{\Phi I}}M_{\Phi I}}{M_{\Phi I} + \eta_{F,M_{\Phi I}}} \right] - k_{lin_F}F_u - k_{b_F}\left((T + I)A_F - F_b\right)F_u + k_{u_F}F_b, \tag{S28}$$

$$\frac{dM}{dt} = \lambda_M \left(M^*_{prod} + \left(\psi_M^{max} - M^*_{prod}\right) \frac{G_b^{h_M}}{G_b^{h_M} + \epsilon_{G,M}^{h_M}} \right) M^*_R + \lambda_M \frac{p_{M,I}I}{I + \epsilon_{I,M}} M - (d_M + \theta_M)M, \tag{S29}$$

$$\frac{dL_u}{dt} = \lambda_{L_u} \left(\frac{p_{L,I}I}{I + \eta_{L,I}} + \frac{p_{L,M}M}{M + \eta_{L,M}} + \frac{p_{L,M_{\Phi I}}M_{\Phi I}}{M_{\Phi I} + \eta_{L,M_{\Phi I}}} \right) - k_{lin_L}L_u - k_{b_L}\left((N + M + T)A_L - L_b\right)L_u + k_{u_L}L_b, \tag{S30}$$

$$\frac{dC_u}{dt} = \lambda_{C_u} \frac{p_{C,M}M}{M + \eta_{C,M}} - k_{lin_C}C_u - k_{b_C}(NA_C - C_b)(C_u)^{POW} + k_{u_C}C_b, \tag{S31}$$

$$\frac{dG_u}{dt} = \lambda_{G_u} \left(\frac{p_{G,M}M}{M + \eta_{G,M}} + \frac{p_{G,M_{\Phi I}}M_{\Phi I}}{M_{\Phi I} + \eta_{G,M_\Phi}} \right) - k_{lin_G}G_u - k_{b_G}(MA_G - G_b)G_u + k_{u_G}G_b (S32)$$

Production deficiencies also modify the initial conditions, as they can affect homeostasis in the absence of infection. Therefore, for all simulations we first allow the immune system to reach homeostasis before challenging the host with the viral inoculum.

2.1. More realistic immunodeficiencies

While we treat immunodeficiencies as a reduction in the production rate of a single variable, clinically observed immunodeficiencies tend to be multifaceted, simultaneously affecting different aspects of the immune response. Four common immunodeficiencies, and their most prominent immunological features, are:

1. **B cell deficiencies and/or lymphomas**: These individuals have a weakened antibody response and may also experience neutrophil deficiencies[10,11] (reduced $A(t)$, $N(t)$), depending on the type of deficiency or lymphoma[12].
2. **Uncontrolled HIV and AIDS**: Individuals with uncontrolled HIV show decreased production of naïve T cells, and CD4+ T cell lymphopenia[13], resulting in a failure to produce antibodies[14]. Further, natural interferon-α producing cells are greatly reduced in individuals with AIDS[15] (reduced $A(t), T(t), F_b(t)$)
3. **Non-B cell cancers**: Individuals with non-B cell malignancies are treated with myelosuppressive chemotherapies that suppress aspects of the innate immune system (e.g., deficiencies in neutrophils and macrophages[16]) as well as CD8+ T cells[17] (reduced $N(t), T(t), M_{\Phi,I}(t)$).
4. **Solid organ transplant recipients**: These individuals are treated with lifelong immunosuppressive drugs[18] and so are deficient in all aspects of the immune response (reduced $A(t), N(t), T(t), M_{\Phi,I}(t)$).

We expect that more realistic immunodeficiencies recapitulate the strongest effects we observed when considering single-variable immunodeficiencies, and so we do not consider them further here.

3. Parameter definitions and parameter values used in simulations

Our immunological model is based off a previous published model[5]; we have made several modifications to this model which we detail here. First, we allow for the reconversion of refractory to susceptible cells after τ_R days in immunocompromised hosts. The rate of this reconversion, δ_R, was taken to be 0.05 days^{-1}, and τ_R was set to 8 days to fall within the range of previous estimates[19].

Second, consistent with studies from the lungs of influenza infected mice[8], we modelled infected cell killing by T cells to be density dependent and saturable using the function

$$\delta_{I,T} \frac{T}{\epsilon_{\delta,T} + I^{h_T}}.$$

By comparing the total number of infected cells in our model versus that of Myers et al.[6], we set the maximal rate of T cell killing to be $\delta_{I,T}$ = 7 days^{-1}, the half-maximal cell concentration, $\epsilon_{\delta,T}$, to be 0.01 x 10^9 cells/mL, and the Hill coefficient, h_T, to be 0.5.

Third, given that primary infections can become protracted in immunocompromised hosts, we included neutralizing antibodies in our model. We assume antibodies are generated by the presence of virions τ_A days post-infection. The delay in antibody production accounts for the time required for the uptake of antigen by antigen presenting cells and their presentation to CD4+ T cells, the initial production of antibodies by short-lived plasma cells after CD4+ T cell priming, and the production of long-lived plasma cells and memory B cells from germinal centres[4]. We assumed antibodies are produced at a constant rate of $p_{A,V} = 500$ days^{-1} and are cleared at rate $d_A = 0.033$ days^{-1}. We model the neutralization of virions by antibodies using the Hill function given by

$$\delta_{V,A} \frac{A^{h_A}}{\epsilon_{V,A}^{h_A} + A^{h_A}}.$$

Based on our previous work where we fit this Hill function to data of SARS-CoV-2 neutralization by antibodies[4,20], we set $\delta_{V,A} = 5$ days^{-1}, $h_A = 1.19$, and $\epsilon_{V,A} = 1000$ AU/mL.

Fourth, we model immunodeficiencies as a reduction in the production of the target variable (see Section 2). In our model, production of cells (i.e., neutrophils, monocytes, macrophages, and T cells) and cytokines (i.e., IFN, G-CSF, GM-CSF, and IL-6) is often generated through multiple pathways (see equations S24-S32). By assuming that immunodeficient hosts had at least a 50% reduction in the target variable compared to healthy hosts, we reduce the rate of production of neutrophils, monocytes, macrophages, IFN, G-CSF, GM-CSF, and IL-6 by 35%. We further reduce T cell and antibody production to more closely resemble clinical observations of lymphopenia[21] and the absence of antibodies in certain patient groups[10,22]. Hence, T cell production is reduced by 75% and antibody production by 90%.

All other parameters in the immunological model were set to their previously estimated values. Full details are provided in the Supplementary Information to Jenner et al.[5]. Briefly, Jenner et al.[5] used a hierarchical estimation procedure in which certain parameter values were fixed directly from the literature, while others were fit using nonlinear least squares or nonlinear mixed effects methods to dose response data or time-series measurements.

In simulations incorporating immunodeficiencies, we calculated any remaining parameter values at physiological homeostasis to ensure that the immunological components return to basal concentrations. In particular, we set the differential equations for the different components of the immune system to 0 (in the absence of the virus), and then we calculated the values of $L_{B,0}$, $C_{B,0}$, $G_{B,0}$, $F_{B,0}$, $M_{\Phi I,0}$, $\epsilon_{L,M_{\Phi I}}$, $\epsilon_{C,M}$, $p_{T,F}$, M^*_{prod}, N^*_{prod}, and $\eta_{F,M_{\Phi I}}$. The complete list of parameter values is provided in Table A.

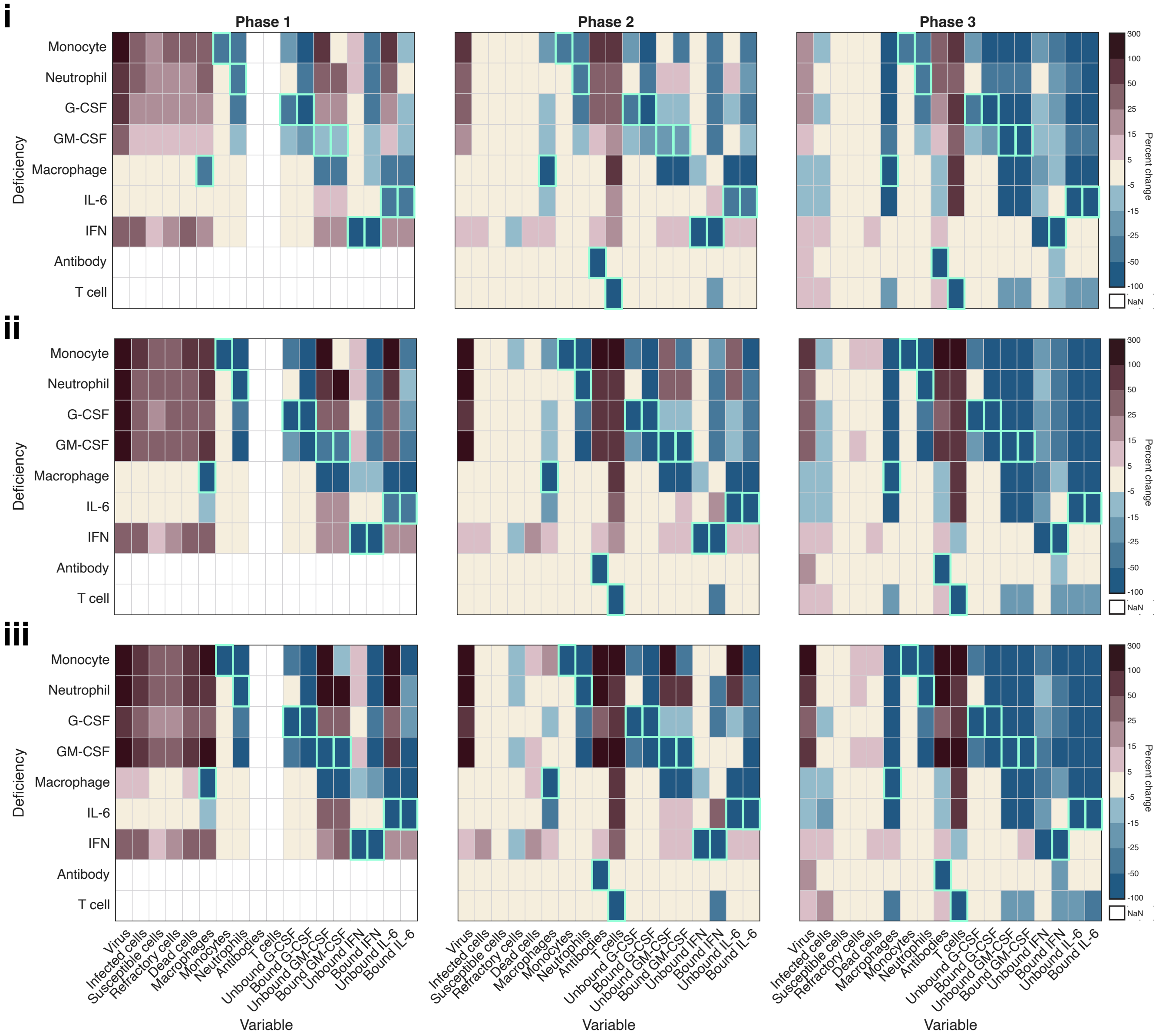

Figure A. Changes in the immunological response in hosts with varying degrees of immunodeficiencies. i-iii) Percent change in each immunological variable (columns) resulting from an immunodeficiency in a key immune cell, cytokine, or antibody (rows) during acute infections (phase one and two; $\delta_R = 0$). The percent change in each immunological variable is calculated as $(X - Y)/X \times 100$, where X and Y are the integrals of the indicated variable over the length of the phase in immunodeficient and healthy hosts, respectively, while the percent change in susceptible cells is calculated using the integral of $S_{max} - S(t)$. Note that NaN values reflect static T cell and antibody responses during the first phase of infection. **i)** Severity of immunodeficiencies reduced by 25% from its baseline value (shown in **ii**). **ii)** Severity of immunodeficiencies used in the main text (baseline value). **iii)** Severity of immunodeficiencies increased by 25% from its baseline value (shown in **ii**). Increasing the severity of the immunodeficiencies results in a quantitative, but not qualitative, change in the concentrations of the different immunological variables.

4. Viral evolution

4.1 Evolutionary model

Next, we extend the immunological model to account for the possibility of viral evolution. To do so, we allow for strains carrying different mutations to simultaneously circulate in the population. We focus throughout on the action of selection and so ignore *de novo* mutations. We therefore assume that the mutant strains under consideration (and so the mutations of interest) are present at low frequencies (specifically, a frequency of 0.01) in the initial viral inoculum.

We consider eight possible mutations. Each of these mutations is beneficial and has a single effect on viral life history. The eight mutations considered are:

1. Virion evasion of antibodies, $A(t)$, through decreased $\delta_{V,A}$.
2. Virion evasion of neutrophils, $N(t)$, through decreased $\delta_{V,N}$.
3. Virion evasion of macrophages, $M_{\Phi,I}(t)$, through decreased $\delta_{V,M}$.
4. Increased virion ability to enter the host cell, $S(t)$, through increased β (e.g., by inhibiting PAMPs[23,24]).
5. Virion evasion of interferon, by reducing the ability of interferon to block viral entry through increased $\epsilon_{F,I}$.
6. Infected cell evasion of T cells, $T(t)$, through decreased $\delta_{I,T}$.
7. Infected cell evasion of neutrophils, $N(t)$, through decreased $\delta_{I,N}$.
8. Infected cell evasion of macrophages, $M_{\Phi,I}(t)$, through decreased $\delta_{I,M}$.

The mutations can be grouped as either "virion mutations", which are mutations that target components of virion fitness (mutations 1-5, that is, decreased $\delta_{V,N}$, $\delta_{V,M}$, $\delta_{V,A}$ or increased β, $\epsilon_{F,I}$), or "infected cell mutations", which are the mutations that target components of infected cell fitness (mutations 6-8, that is, decreased $\delta_{I,N}$, $\delta_{I,M}$, $\delta_{I,T}$). Note that $\epsilon_{F,I}$ is classified here as a "virion mutation" mainly because the eclipse phase is short, and so it is more closely linked to virion fitness than infected cell fitness.

For each mutation, we divide the immunological variables into the variable directly targeted by the mutation (henceforth the "target") and all other immunological variables not targeted by the focal mutation but targeted by a different mutation (henceforth, "nontargets"). For example, if the focal mutation targets virion evasion of neutrophils (decreased $\delta_{V,N}$), then the target variable is neutrophils, while the nontarget variables are macrophage, interferon, antibodies, T cells, and susceptible cells.

Next, let $I_i(t)$ and $V_i(t)$ denote the density of cells infected with strain i and strain i virions, respectively. Strain i has phenotype $\{\delta_{V,N}^i, \delta_{V,M}^i, \delta_{V,A}^i, \beta^i, \epsilon_{F,I}^i, \delta_{I,N}^i, \delta_{I,M}^i, \delta_{I,T}^i\}$. Then we can write the dynamics of strain i as

$$\frac{dV_i}{dt} = pI_i - \overbrace{\left(d_V + \delta_{V,N}^i N + \delta_{V,M}^i M_{\Phi,I} + \frac{\delta_{V,A}^i A^{h_A}}{\left(\epsilon_{V,A}^{h_A} + A^{h_A}\right)}\right)}^{m_V^i} V_i, \quad (S32)$$

$$\frac{dI_i}{dt} = \underbrace{\frac{\beta^i \epsilon_{F,I}^i}{\epsilon_{F,I}^i + F_b} S(t-\tau_I)}_{B_I^i} V_i(t-\tau_I) - \underbrace{\left(d_I + \frac{\delta_{I,N}^i N^{h_N}}{N^{h_N} + IC_{50,N}^{h_N}} + \delta_{I,M}^i M_{\Phi,I} + \frac{\delta_{I,T} T}{\epsilon_{\delta,T} + I^{h_T}}\right)}_{m_I^i} I_i. \quad (S33)$$

where B_I^i is the per-capita rate at which strain i virions produce cells infected by strain i, while m_V^i and m_I^i are the per-capita decay rates of strain i virions and infected cells, respectively.

4.2 Evolution of individual mutations

We first consider the evolution of each individual mutation. To do so, we note that if $p_x(t)$ denotes the frequency of mutation x within the viral population, the frequency of mutation x changes according to the equation

$$\frac{dp_x}{dt} = s_x(t) p_x (1 - p_x), \quad (S34)$$

where $s_x(t)$ is the time-varying selection coefficient. The strength of selection on mutation x at time t is determined by the magnitude of $s_x(t)$; this dictates the instantaneous rate of increase of the mutation, given an observed mutation frequency. In turn, $s_x(t)$ can be used to calculate the time-averaged strength of selection on mutation x from time t_0 to time t:

$$\langle s_x(t) \rangle = \frac{1}{t - t_0} \int_{t_0}^{t} s_x(\tau) d\tau. \quad (S35)$$

Equation (S35) measures how strong (constant) selection would have to be to yield the observed change in frequency by time t.

Thus, $s_x(t)$ and $\langle s_x(t) \rangle$ capture how the immune response determines the short- and long-term strength of selection, respectively. This in turn dictates the speed of adaptation and so will be our focus here. Specifically, we will use $s_x(t)$ and $\langle s_x(t) \rangle$ to understand how immunodeficiencies affect the speed of evolution of different mutations targeting viral evasion of the immune response.

4.2.1 Approximating per-capita growth rate

To calculate the selection coefficient, we need to compute the per-capita growth rates, r_i, of the different strains. This is because if there are two strains, $i \in \{x_w, x_m\}$, differing based on a single mutation, then the selection coefficient acting on mutation x is

$$s_x(t) = r_{x_m}(t) - r_{x_w}(t). \quad (S36)$$

There are two challenges to computing per-capita growth rate in our model. The first is the presence of the delay due to eclipse time, τ_I. To remove the delay, define $E_i(t)$ to be the density of cells that have been infected by strain i, but are not yet productively infected. Then we can apply the approximation

$$\frac{dV_i}{dt} = pI_i - m_V^i V_i, \tag{S37}$$

$$\frac{dE_i}{dt} = \beta^i S V_i - \frac{1}{\tau_I} E_i, \tag{S38}$$

$$\frac{dI_i}{dt} = \frac{1}{\tau_I} \frac{\epsilon_{F,I}^i}{\epsilon_{F,I}^i + F_b} E_i - m_I^i I_i. \tag{S39}$$

The second challenge to calculating per-capita growth rate arises due to the fact individuals belong to different classes (i.e., virions, infected cells, and productively infected cells). Calculating per-capita growth rates in populations where individuals belong to different classes (i.e., virions and infected cells) is not trivial, particularly for populations with temporally varying per-capita growth rates[25-28]. Therefore, to gain some analytic insight into how immunodeficiencies affect the strength of selection, suppose the immunological variables, and density of susceptible cells, change slowly relative to the change in infected cells and virions (e.g., see Day et al. 2022[27]). Then the per-capita growth rate of strain i is the dominant eigenvalue, r_i, of the matrix

$$R_i = \begin{pmatrix} -m_V^i & 0 & p \\ \beta^i S & -\frac{1}{\tau_I} & 0 \\ 0 & \frac{1}{\tau_I} \frac{\epsilon_{F,I}^i}{\epsilon_{F,I}^i + F_b} & -m_I^i \end{pmatrix}. \tag{S40}$$

Because R_i is a 3×3 matrix, finding the dominant eigenvalue means finding the roots of the cubic polynomial:

$$\lambda^3 + \left(m_I^i + m_V^i + \frac{1}{\tau_I}\right)\lambda^2 + \left(m_I^i m_V^i + \frac{m_V^i + m_I^i}{\tau_I}\right)\lambda + \frac{m_I^i m_V^i}{\tau_I} - \frac{p}{\tau_I} \frac{\beta^i S \epsilon_{F,I}^i}{\epsilon_{F,I}^i + F_b}.$$

This is numerically straightforward but poses a challenge analytically. However, as the eclipse phase is short (τ_I is small), it is not unreasonable to suppose that the dynamics of non-productively infected cells occurs on a fast time scale, relative to the dynamics of virions and productively infected cells, that is $dE_i/dt \approx 0$, and so on the slow scale

$$E_i(t) \approx \frac{\beta^i S V_i}{\tau_I}$$

This approximation yields the reduced system on the slow time scale,

$$\frac{dV_i}{dt} = pI_i - m_V V_i \tag{S41}$$

$$\frac{dI_i}{dt} = \beta^i S \frac{\epsilon_{F,I}^i}{\epsilon_{F,I}^i + F_B} V_i - m_I^i I_i = B_I^i V_i - m_I^i I_i, \tag{S42}$$

where

$$B_I^i = \beta^i S \frac{\epsilon_{F,I}^i}{\epsilon_{F,I}^i + F_B}.$$

For the reduced system, the per-capita growth rate of strain i is the dominant eigenvalue, r_i, of the matrix

$$R_i = \begin{pmatrix} -m_V^i & p \\ B_I^i & -m_I^i \end{pmatrix}. \tag{S43}$$

Thus

$$r_i = \frac{\theta^i - m_V^i - m_I^i}{2} \tag{S44}$$

where $\theta^i = \sqrt{4B_I^i p + (\bar{m}^i)^2}$ and $\bar{m}^i \equiv m_V^i - m_I^i$. Numerical results indicate that approximating r_i using equation (S44) and using this to calculate the selection coefficient, s_x, is very good, with the only deviation occurring at the beginning of the infection (Fig. B).

For the numerical results presented in the main text, s_x can be approximated using simulation data as

$$s_x(t) \approx -\frac{1}{t_i - t_{i-1}} \ln\left(\frac{p_x^I(t_{i-1})\big(1 - p_x^I(t_i)\big)}{p_x^I(t_i)\big(1 - p_x^I(t_{i-1})\big)}\right), \tag{S45}$$

where $p_x^I(t)$ is the frequency of mutation x at time t in infected cells, and $t_i - t_{i-1}$ is some (short) increment of time (e.g., hours, days). $\langle s_x(t) \rangle$ can be approximated using equation (S45) by setting $t_{i-1} = t_0$.

Although equation (S45) only considers the frequency of the mutation in infected cells and while the "true" selection coefficient involves a weighted average of the frequencies of virions and infected cells, simulation results indicate that it largely yields similar predictions if it is calculated using the frequency of the mutation in virions (Fig. B). The principal area of divergence between the different measures occurs at the onset of the infection. This is because each infection starts with only virions present, and so the delay in production of infected cells due to the eclipse phase means equation (S45) cannot be accurately calculated during the initial few hours of infection (Fig. B). Moreover, the calculations of the selection coefficient using eigenvalues capture the strength of selection after the initial transient dynamics owing to the initial conditions are finished. Therefore, in all simulation results in the main text we plot equation (S45) from hour 12 onwards.

The other key point from Figure B is that mutations affecting virion and infected cell evasion of macrophage, and mutations targeting infected cell evasion of neutrophils are under very weak selection (Fig. B vi-viii). Specifically, the time for a mutation with a constant selection coefficient, s, to increase in frequency from p_0 to p_1 is

$$t = -\frac{1}{s} \ln\left(\frac{p_0(1 - p_1)}{p_1(1 - p_0)}\right).$$

The time-averaged selection coefficient for mutations affecting evasion of macrophage and infected cell evasion of neutrophils are on the order of 10^{-3} or less, and so using the above equation it would take approximately 9,190 days for a mutation to increase from a frequency of $p_0 = 0.01$ to $p_1 = 0.99$. This very weak selection remains true regardless of the size of the

mutational effect; indeed, in Figure B vii-viii, the mutational effects for virion and infected cell evasion of macrophage, and infected cell evasion of neutrophils, are maximal.

The immunological reasoning for the negligible selection on mutations affecting $\delta_{V,M}$, $\delta_{I,M}$, and $\delta_{I,N}$ is as follows. The primary role of neutrophils during viral infections is to neutralize virions[29] and not infected cells. Consequently, neutrophils have a saturated killing rate of infected cells[5], which translates to weak selection on infected cell evasion of neutrophils. Macrophages, on the other hand, have two key effects. First, they balance inflammation and tissue repair, helping bridge the innate and adaptive immune responses[30]. Second, they clear pathogenic and cellular debris[31] through phagocytosis once cells have become damaged or die. While the first effect is important for immune system functioning, it will not select for macrophage evasion, and the second effect occurs near the end of the infected cells life and so has limited selective consequences.

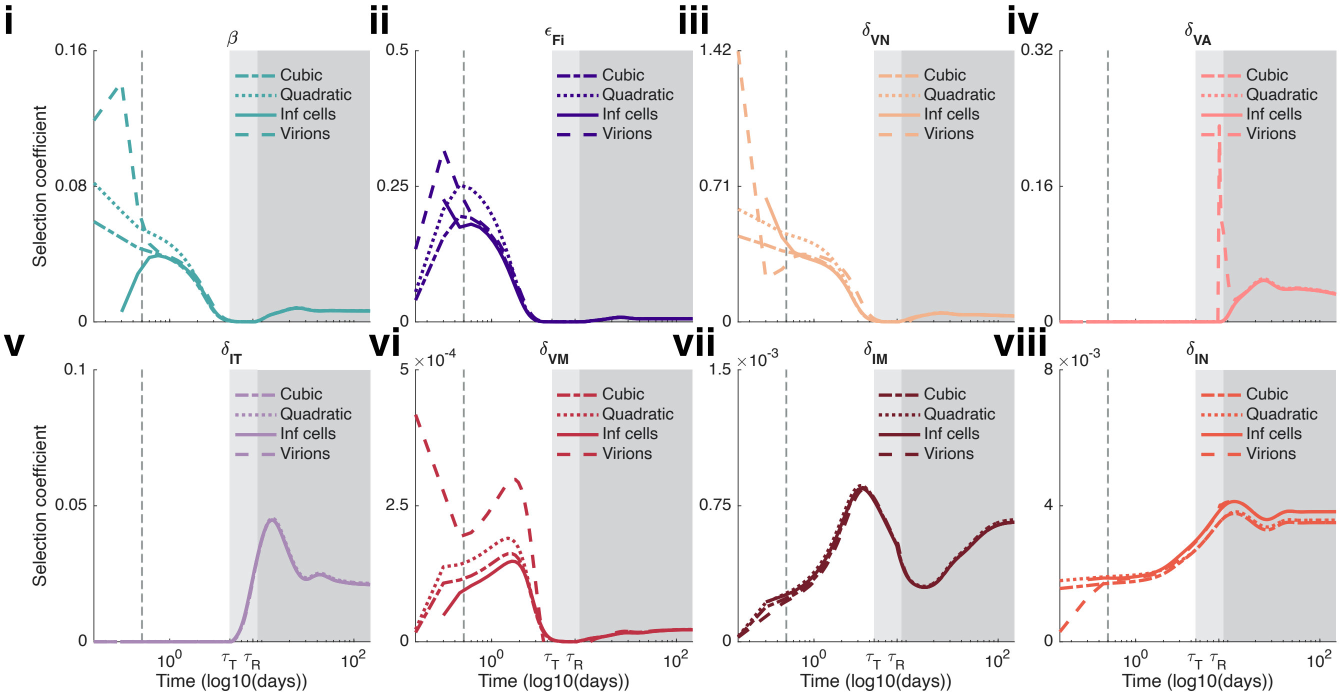

Figure B. Comparison of different approximations of the selection coefficient. Selection coefficients numerically calculated four ways: (1) using formula (S36), with r_{x_m} and r_{x_w} calculated using the matrix in equation (S40) (dashed-dot lines); (2) using formula (S36), with r_{x_m} and r_{x_w} given by equation (S44) (dotted lines); (3) applying equation (S45) to simulation data (solid lines); or (4) applying equation (S45) to simulation data, except by measuring frequency amongst virions (dashed lines) for **i)** β, **ii)** ϵ_{FI}, **iii)** δ_{VN}, **iv)** δ_{VA}, **v)** δ_{IT}, **vi)** δ_{VM}, **vii)** δ_{IM}, and **viii)** δ_{IN} mutations. Virion and infected cell evasion of macrophage (**vi** and **vii**), and infected cell evasion of neutrophils (**viii**) are under very weak selection, even though the mutational effect size is maximal. **i-viii)** Dashed vertical grey line: 12 hours post-infection. White background: first phase of infection. Light grey background: second phase of infection. Dark grey background: third phase of infection.

4.2.2 Weak selection approximation

We can gain further insight into the selection coefficient by supposing the mutational difference between the wildtype and mutant phenotypes, $\Delta x \equiv x_m - x_w$, is small, that is, selection is weak. Then we can use r_i to approximate s_x as

$$s_x \approx \left(-\frac{1}{2}\left(1-\frac{\bar{m}^w}{\theta^w}\right)\frac{\partial m_V^w}{\partial x_w}-\frac{1}{2}\left(1+\frac{\bar{m}^w}{\theta^w}\right)\frac{\partial m_I^w}{\partial x_w}+\frac{pB_I^w}{\theta^w}\frac{\partial f_x^w}{\partial x_w}\right)\Delta x+\mathcal{O}(\Delta x^2), \qquad (S46)$$

where $\partial f^w/\partial x_w = 1$ if $x = \beta$ and $\partial f^w/\partial x_w = F_b/(\epsilon_{F,I} + F_b)$ if $x = \epsilon_{F,I}$.

Note that each of the $\partial z/\partial x_w$ are non-negative for $z \in \{m_V^w, m_I^w, f^w\}$, while $\Delta x < 0$ if mutation x affects the destruction of virions (m_V) or infected cells (m_I), whereas $\Delta x > 0$ if mutation x targets the infection of susceptible cells (B_I). Consequently, each term in equation (S46) is positive, as observed in the main text.

From our approximation of s_x, the strength of selection (magnitude of s_x), depends on two factors. The first factor is the direct impact of mutation x on the relevant viral life-history quantity (i.e., the magnitude of $\frac{\partial z}{\partial x_w}\Delta x$, for $z \in \{m_V, m_I, f\}$. Since each mutation has a single effect on viral life history, only one direct effect will be non-zero. The second factor is how the direct effect is weighted due to class-structure (e.g., the magnitude of $(1/2)(1-\bar{m}^w/\theta^w)$). These weights capture the distribution of mutations between the different classes, as well as the reproductive value of each class.

In the first phase of the infection, s_x is as given in equation (S45). In the second phase of infection, susceptible cells have been largely depleted, that is, $S(t) \approx 0$, and so $B_I^w \approx 0$. Consequently, $\theta^w \approx m_V^w - m_I^w$ (since $m_V^w > m_I^w$), and so equation (S46) reduces to

$$s_x \approx -\frac{\partial m_I}{\partial x}\Delta x. \qquad (S47)$$

Thus, the value of virion mutations is zero, while the value of infected cell mutations is at its maximum. In the third phase of infection, the concentration of virions and infected cells will eventually be in a quasi-equilibrium state. Therefore, we have

$$0 \approx pI - m_V V \quad \text{and} \quad 0 \approx B_I V - m_I I,$$

which implies that

$$\frac{V}{I} \approx \frac{p}{m_V} \approx \frac{m_I}{B_I}$$

or $pB_I \approx m_V m_I$. Consequently, $\theta \approx m_V + m_I$, and so equation (S46) reduces to

$$s_x \approx \left(-\frac{m_I^w}{m_I^w+m_V^w}\frac{\partial m_V^w}{\partial x_w}-\frac{m_V^w}{m_I+m_V^w}\frac{\partial m_I^w}{\partial x_w}+\frac{m_V^w m_I^w}{m_I^w+m_V^w}\frac{\partial f_x^w}{\partial x_w}\right)\Delta x+\mathcal{O}(\Delta x^2). \qquad (S48)$$

Because $m_V^w > m_I^w$, the value of mutations targeting infected cell evasion of the immune response is higher than the value of mutations targeting virion evasion of the immune response.

4.3 Similar evolutionary dynamics are observed for different immunodeficiencies

In the main text, we observed that certain immunodeficiencies have qualitatively similar effects on the strength of selection; this is shown in Figure C. Immunodeficiencies in neutrophils, monocytes, and/or the cytokines G-CSF and GM-CSF induce qualitatively similar evolutionary dynamics (Fig. C i-iv). This is because each of these deficiencies trigger a reduction in neutrophil concentrations (Fig. A). Similarly, immunodeficiencies in IL-6 and macrophage overstimulate T cell concentrations and so induce qualitatively similar evolutionary dynamics during persistent infections (Fig. C v-vi). T cell deficiencies and IFN deficiencies, on the other hand, yield distinct evolutionary dynamics (Fig. C vii-viii).

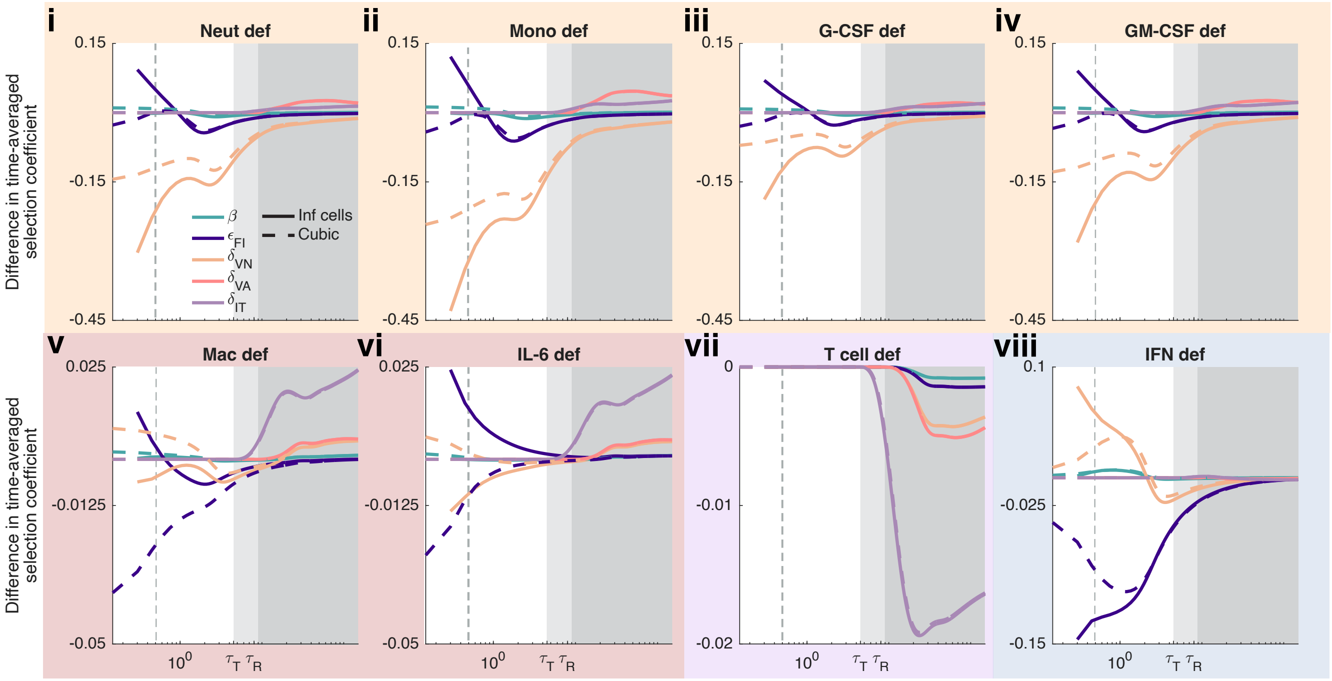

Figure C. Qualitative similarities in evolutionary dynamics are determined by the type of immunodeficiency. Time-averaged selection coefficient calculated using formula (S36), with r_{x_m} and r_{x_w} calculated as the dominant eigenvalue of matrix (Eq. S40) (dashed lines), or by applying equation (Eq. S45) to simulation data (solid lines). Qualitatively similar viral evolutionary dynamics emerge for **i)** neutrophil, **ii)** monocyte, **iii)** G-CSF, and **iv)** GM-CSF deficiencies (orange background), **v)** macrophage and **vi)** IL-6 deficiencies (red background), while **vii)** T cell (purple background) and **viii)** IFN (blue background) deficiencies show distinct dynamics. The divergence between these measures at the beginning of the infection arises due to two sources: (1) estimating the selection coefficient from the simulation data only takes into account the frequency of mutations in infected cells, whereas the true selection coefficient is a weighted average of infected cells and virions, and (2) at the beginning of the infection, there are only virions present and there is a delay owing to the eclipse phase before infected cells appear. **i-viii)** Dashed vertical grey line: 12 hours post-infection. White background: first phase of infection. Light grey background: second phase of infection. Dark grey background: third phase of infection.

4.4 Effect of inoculum size on infection dynamics

All infections initially consist of some concentration of virions, $V(0) > 0$, and no infected cells, $I(0) = 0$. However, we would expect some heterogeneity in the size of the initial inoculum across hosts. Heterogeneity in initial inoculum size affects the duration of phases of infection as well as the strength of the immune response. A smaller inoculum size slows the growth of the initial infection, slowing the depletion of susceptible cells and so extending the first phase of infection (Fig. D). This will also tend to weaken the immune response across the first two phases of infection. The inoculum size has negligible consequences during the third phase of infection. Simulation results show that an inoculum size of at least $V(0) = 10^{-4}$ log(copies/mL) is required to initiate an infection.

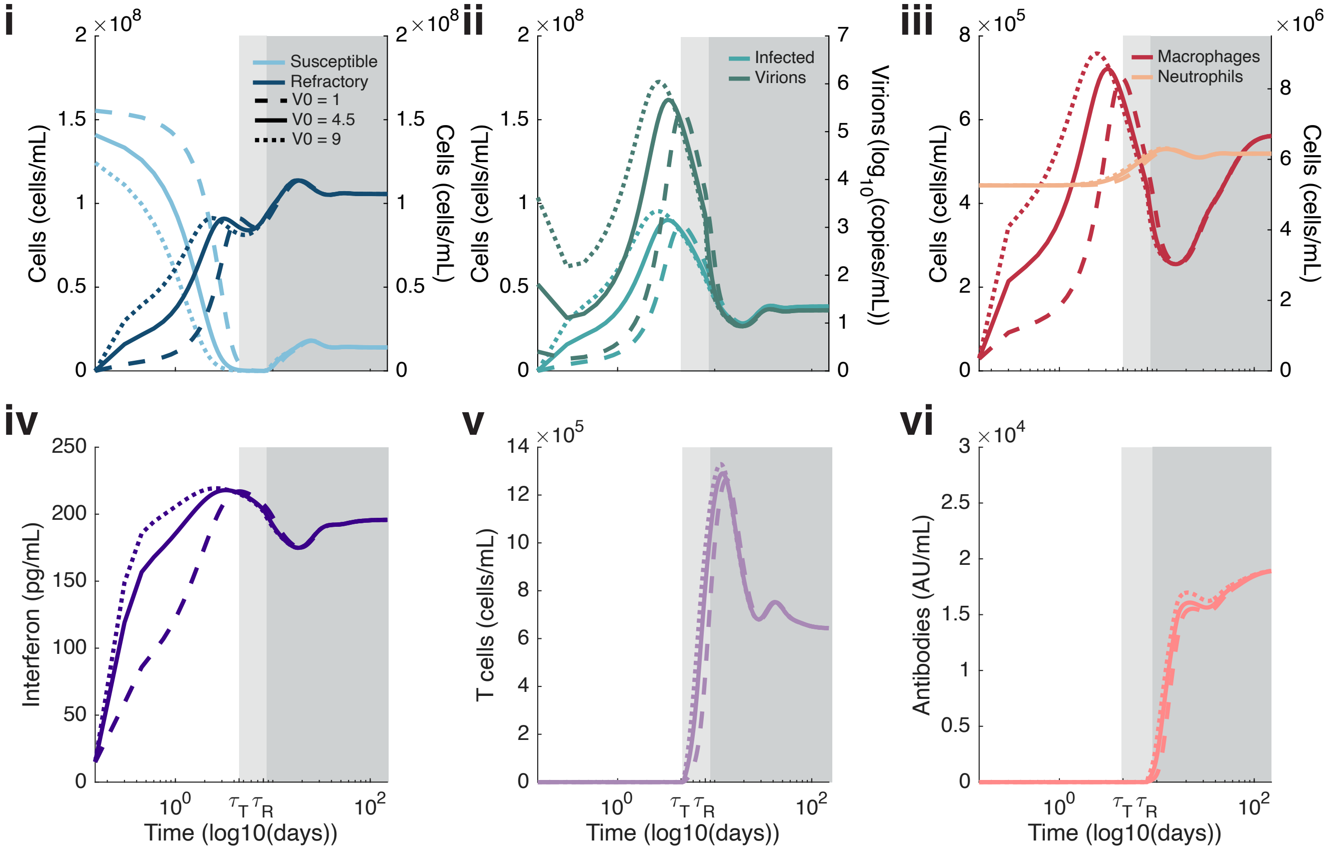

Figure D. Inoculum size influences the onset of infection phases. Dynamics of **i)** susceptible and refractory cells, **ii)** infected cells and virions, **iii)** macrophages and neutrophils, **iv)** interferon, **v)** T cells, and **vi)** antibodies for initial viral inoculum size of 1, 4.5, and 9 $\log_{10}$(copies/mL). Larger inoculum sizes shorten (shift left) the first phase of infection due to higher viral loads and faster depletion of susceptible cells, while the reverse is true for smaller inoculum sizes. **i-vi)** White background: first phase of infection; light grey background: second phase of infection; dark grey background: third phase of infection regions for an inoculum of 4.5 $\log_{10}$(copies/mL), as in the results shown in the main text.

4.5 Multiple mutations

Next, we consider the evolution of multiple mutations. To do so, we suppose there are two mutations, x and y, at different loci affecting different aspects of the viral life history. This means there are four pathogen strains to consider, $i \in \{x_w y_w, x_m y_w, x_w y_m, x_m y_m\}$.

When mutations at different loci are simultaneously segregating in the population, in addition to selection on each individual mutation, we also need to account for epistasis in fitness between mutations. In continuous time models, if r_i is the per-capita growth rate of strain i, then epistasis in fitness is defined as

$$s_{xy} \equiv r_{x_m y_m} + r_{x_w y_w} - r_{x_w y_m} - r_{x_m y_w}. \tag{S49}$$

Thus, epistasis in fitness captures whether the fitness of a mutation depends on the genetic background or not.

If epistasis in fitness is positive, strains carrying both mutations are fitter than would be expected, based upon the fitness contribution of each individual mutation, speeding their evolution. If epistasis in fitness is negative, strains carrying both mutations are less fit than expected, slowing their evolution (in extreme cases, this can cause individually beneficial mutations to decrease in frequency). In addition, epistasis in fitness can produce linkage disequilibrium (LD) between mutations. The presence of LD, or the non-random associations between mutations, means that selection on mutation y_m will cause changes in the frequency of mutation x_m (so-called indirect selection). Because both mutations are individually beneficial, if epistasis is negative, then indirect selection will slow the evolution of both mutations, whereas if epistasis is positive, then indirect selection will speed the evolution of both mutations. Thus, in our model, indirect selection, and the direct consequences of epistasis work together to speed (positive epistasis) or slow (negative epistasis) evolution.

4.5.1 Approximating epistasis in fitness

As before, per-capita growth rates are difficult to calculate in class-structured populations. Therefore, to make analytic progress we apply the same approximation as before. We assume that immunological variables, and density of susceptible cells, change slowly relative to the change in infected cells and virions and that the eclipse phase is short. Then under weak selection (i.e., the differences $x_m - x_w$ and $y_m - y_w$ are small), a Taylor series expansion of s_{xy} to leading order can be written as

$$s_{xy} \approx \frac{pB_I^w}{(\theta^w)^3}\left(\left(\frac{\partial m_V^w}{\partial x} - \frac{\partial m_I^w}{\partial x}\right)\left(2\frac{\partial m_V^w}{\partial y} + \bar{m}^w \frac{\partial f^y}{\partial y}\right) + \frac{\partial m_I^w}{\partial x}\frac{\partial m_I^w}{\partial y}\right)|\Delta x||\Delta y| + \frac{pB_I^w}{(\theta^w)^3}\left(2pB_I^w + (\bar{m}^w)^2\right)\frac{\partial f^x}{\partial x}\frac{\partial f^y}{\partial y}|\Delta x||\Delta y|\,, \tag{S50}$$

where $\partial f^z/\partial z = 1$ if the mutation z affects transmissibility, β, and $\partial f^z/\partial z = F_b/(\epsilon_{F,I} + F_b)$ if the mutation z affects evasion of IFN. As each mutation has a single direct effect, only one of the $\partial q/\partial x$ and one of the $\partial q/\partial y$ are non-zero for $q \in \{m_V, m_I, f\}$.

4.5.2 The sign of epistasis in fitness

In our model, epistasis in fitness comes from two sources. First, if the two mutations target infection of susceptible cells (increased β) and interferon evasion (increased $\epsilon_{F,I}$), these two terms multiplicatively affect the production of infected cells. This produces a positive epistatic interaction affecting B_I that will ultimately translate to positive epistasis in fitness. Second, class

structure means that even in the absence of epistatic interactions affecting one component of viral life history, epistasis in fitness tends to be generated[32].

Recall that we previously identified two groups of mutations based on their direct fitness effects. In the first group are virion mutations. These mutations target virion infection and entry into cells (i.e., increased β, $\epsilon_{F,I}$) or target virion evasion of the immune response (i.e., decreased $\delta_{V,A}$, $\delta_{V,N}$, $\delta_{V,M}$). In the second group are infected cell mutations. These mutations target infected cell evasion of the immune response (i.e., decreased $\delta_{I,T}$, $\delta_{I,N}$, $\delta_{I,M}$). Our approximation of s_{xy} makes two predictions concerning these groups. If both mutations belong to the same group, epistasis in fitness is positive. If one mutation belongs to one group, and the other mutation belongs to the other group, epistasis in fitness is negative.

Negative epistasis is weak in our model. This occurs because the per-capita decay rate of virions is higher than the per-capita decay rate of infected cells. Consider the case in which $m_V^i \gg m_I^i$. Then we can approximate the density of virions of strain i as

$$V_i \approx \frac{p}{m_V^i} I_i. \tag{S51}$$

Using this relationship, we have

$$\frac{dI_i}{dt} \approx \left(\frac{B_I^i p}{m_V^i} - m_I^i\right) I_i, \tag{S52}$$

and so

$$r_i \approx \frac{B_I^i p}{m_V^i} - m_I^i. \tag{S53}$$

If we use this per-capita growth rate in the formula for epistasis given in equation (S49), we can see that negative epistasis is not possible between pairs of beneficial mutations.

4.6 Parameter values

Parameter	Units	Description	Value	Source
		Viral kinetic parameters		
p	1/day ×log(cop/ml) /10^9cells	Lytic viral production	2.59	5
κ_S	1/day	Proliferation of epithelial cells	0.7397	5
S_{max}	10^9cells	Epithelial cells carrying capacity	S_0	5
$k_{M\Phi}$	log(cop/ml)/day	Production of alveolar macrophages	5943	5
$M_{\Phi max}$	10^9cells/ml	Alveolar macrophage carrying capacity	$M_{\Phi R,0}$	5
β	1/day× 1/ log(cop/ml)	SARS-CoV-2 virus infection rate	0.30	5
τ_I	days	Eclipse time	0.17	5
τ_T	days	Delay in CD8+ T cell arrival	4.5	5

Parameter	Units	Description	Value	Source
τ_R	days	Delay in refractory cell re-entry	8	19
τ_A	days	Delay in antibody production	5	4
		Cell production, recruitment, and activation rates		
$p_{M\Phi I,G}$	1/day	Monocyte to macrophage differentiation by GM-CSF	1.68	5
$p_{M\Phi I,L}$	1/day	Monocyte to macrophage differentiation by IL-6	1.68	5
$a_{I,M\Phi}$	ml/(10^9cells)× (1/day)	Activation of macs by infected and dead cells	1.1×10^3	5
$p_{M,I}$	1/day	Monocyte recruitment rate by infected cells	0.22	5
$p_{T,F}$	1/day	CD8+ T cell production rate by IFN	4	5
$p_{N,L}$	1/day	Neutrophils recruitment rate by IL-6	0.3	5
$p_{T,I}$	1/day	CD8+ T cell proliferation rate	0.02	5
$p_{A,V}$	1/day	Antibody production by virus	500	Fixed based on 4,20
M^*_{prod}	1/day	Homeostasis reservoir release rate	0.13	5
ψ^{max}_M	1/day	Maximal reservoir release rate	11.55	5
N^*_{prod}	1/day	Homeostasis reservoir release rate	0.21	5
ψ^{max}_N	1/day	Maximal reservoir release rate	4.13	5
C^*_{bF}	Dimensionless	Homeostasis neutrophil receptor bound fraction	1.6×10^{-5}	5
		Cell-related half-effect (ϵ), IC50 (IC_{50}), and Hill coefficient (h) parameters		
$\epsilon_{F,I}$	pg/ml	IFN inhibition of viral production	2×10^{-4}	5
$\epsilon_{L,M\Phi}$	pg/ml	IL-6 monocytes to macrophages	1102.9	5
$\epsilon_{G,M\Phi I}$	pg/ml	GM-CSF monocyte to macrophages	2664.5	5
$\epsilon_{G,M}$	pg/ml	GM-CSF recruitment of monocytes	57.2	5
$\epsilon_{F,T}$	pg/ml	IFN production of CD8+ T cells	399.3	5
$\epsilon_{C,N}$	unitless	G-CSF recruitment of neutrophils	1.89×10^{-4}	5
$\epsilon_{L,N}$	pg/ml	IL-6 recruitment of neutrophils	57.2	5
$\epsilon_{I,M}$	10^9cells/ml	Infected cell monocyte recruitment	0.05	5
$\epsilon_{L,T}$	pg/ml	IL-6 production of CD8+ T cells	1.5×10^{-5}	5
$\epsilon_{V,M\Phi}$	log(cop/ml)	Viral load for mac replenishing	905.22	5
$\epsilon_{\delta,T}$	10^9 cells/ml	Killing of infected cells by CD8+ T cells	0.01	Recalculated from 6

Parameter	Units	Description	Value	Source
$\epsilon_{V,A}$	AU/ml	Antibody neutralization	1000	4,20
h_M	Dimensionless	GM-CSF monocyte recruitment	1.67	5
$h_{M,M\Phi}$	Dimensionless	GM-CSF monocyte to macrophages	2.03	5
h_N	Dimensionless	Neutrophil induced damage	3.02	5
h_A	Dimensionless	Antibody neutralization	1.19	4,20
h_T	Dimensionless	Killing of infected cells by CD8+ T cells	0.5	Recalculated from 6
$IC_{50,N}$	10^9 cells/ml	Neutrophil induced damage	4.7×10^{-5}	5
		Cell/virus-induced death rates		
$\delta_{V,M\Phi}$	ml/(10^9cells)× 1/day	Rate of viral clearance by macrophages	768	5
$\delta_{V,N}$	ml/(10^9cells)× 1/day	Rate of viral clearance by neutrophils	1152	5
δ_N	1/day	Rate of neutrophil inflicted damage	1.68	5
ρ	Dimensionless	Bystander death modulation constant	0.5	5
$\delta_{I,M\Phi}$	ml/(10^9cells)× (1/day)	Rate macrophages phagocytose infected cells	121.20	5
$\delta_{I,T}$	ml/(10^9cells)× (1/day)	Rate CD8+ T cells induce apoptosis in infected cells	7	Recalculated from 6
$\delta_{M\Phi,D}$	ml/(10^9cells)× (1/day)	Rate macrophages die from phagocytosis	6.06	5
$\delta_{D,M\Phi}$	ml/(10^9cells)× (1/day)	Rate macrophages phagocytose dead cells	8.03	5
δ_R	1/day	Rate of refractory cell reconversion	0 (Immunocompetent)<br>0.05 (Immunodeficiency)	19
$\delta_{V,A}$	log(copies/ml) x (1/day)	Rate of antibody neutralization	5	4,20
		Cell death and decay rates		
d_V	1/day	Viral decay rate	1.81	5
d_I	1/day	Infected cell death rate	0.1	5
d_D	1/day	Degradation rate of apoptosed cells	8	5
$d_{M\Phi R}$	1/day	Alveolar macrophage death rate	0.01	5
$d_{M\Phi I}$	1/day	Inflammatory macrophage death rate	0.3	5
d_M	1/day	Monocyte death rate	0.76	5
d_N	1/day	Neutrophil death rate	0.4	5
d_T	1/day	CD8+ T cell death rate	0.4	5
d_A	1/day	Antibody clearance rate	0.033	4,20
		Cytokine production rates		
$p_{L,I}$	pg/ml/day	IL-6 production by infected cells	11.89	5
$p_{L,M\Phi I}$	pg/ml/day	IL-6 production by inflammatory macrophages	1872	5
$p_{L,M}$	pg/ml/day	IL-6 production by monocytes	72.56	5
$p_{G,M\Phi I}$	pg/ml/day	GM-CSF production by inflammatory macrophages	2626	5

Parameter	Units	Description	Value	Source
$p_{C,M}$	ng/ml/day	G-CSF production by monocytes	26.26	5
$p_{G,M}$	pg/ml/day	GM-CSF production by monocytes cells	123.4	5
$p_{F,I}$	pg/ml/day	IFN production by infected cells	2.82	5
$p_{F,M_{\Phi I}}$	pg/ml/day	IFN production by inflammatory macrophages	1.3	5
$p_{F,M}$	pg/ml/day	IFN production by monocytes	3.56	5
		Half-effect parameters		
$\eta_{L,I}$	10^9 cells/ml	IL-6 production by infected cells	0.7	5
$\eta_{L,M}$	10^9 cells/ml	IL-6 production by monocytes	0.0045	5
$\eta_{L,M_{\Phi I}}$	10^9 cells/ml	IL-6 production by inflammatory macrophages	3.6×10^{-5}	5
$\eta_{G,M\Phi}$	10^9 cells/ml	GM-CSF production by macrophages	3.6×10^{-5}	5
$\eta_{G,M}$	10^9 cells/ml	GM-CSF production by monocytes	0.15	5
$\eta_{C,M}$	10^9 cells/ml	G-CSF production by monocytes	3.05	5
$\eta_{F,I}$	10^9 cells/ml	IFN production by infected cells	0.011	5
$\eta_{F,M_{\Phi I}}$	10^9 cells/ml	IFN production by inflammatory macrophages	1×10^{-5}	5
$\eta_{F,M}$	10^9cells/ml	IFN production by monocytes	0.54	5
		Cytokine clearance and internalization rates		
k_{lin_L}	1/day	Rate of IL-6 renal clearance	16.6	5
k_{lin_G}	1/day	Rate of GM-CSF renal clearance	11.7	5
k_{lin_C}	1/day	Rate of G-CSF renal clearance	0.16	5
k_{lin_F}	1/day	Rate of IFN renal clearance	18	5
k_{in_L}	1/day	Internalization rate of IL-6	61.8	5
k_{in_G}	1/day	Internalization rate of GM-CSF	73.4	5
k_{in_C}	1/day	Internalization rate of G-CSF	462	5
k_{in_F}	1/day	Internalization rate of IFN	17	5
		Cytokine binding/unbinding rates and stoichiometric constant		
k_{b_L}	ml/pg/day	IL-6 binding rate	0.0018	5
k_{b_G}	ml/pg/day	GM-CSF binding rate	0.0021	5
k_{b_C}	ml/ng/day	G-CSF binding rate	2.24	5
k_{b_F}	ml/pg/day	IFN binding rate	0.011	5
k_{u_L}	1/day	IL-6 unbinding rate	22.3	5
k_{u_G}	1/day	GM-CSF unbinding rate	522	5
k_{u_C}	1/day	G-CSF unbinding rate	184	5
k_{u_F}	1/day	IFN unbinding rate	6.07	5
POW	Dimensionless	Stoichiometric constant (G-CSF)	1.4608	5

Parameter	Units	Description	Value	Source
$\hat{p}$	Dimensionless	Stoichiometric constant (IL-6, GM-CSF, IFN)	1	5
		Stoichiometry relating constant (G-CSF)	1.46	5
		Stoichiometry relating constant (IL-6, GM-CSF, IFN)	1	5
		Number of cellular receptors and cytokine molecular weights		
$K_{L,N}$	sites/cell	No. IL-6 receptors on neutrophils	720	5
$K_{L,T}$	sites/cell	No. IL-6 receptors on T cells	300	5
$K_{L,M}$	sites/cell	No. of IL-6 receptors on monocytes	509	5
$K_{G,M}$	sites/cell	No. GM-CSF receptors on monocyte	1058	5
$K_{C,N}$	sites/cell	No. of G-CSF receptors on neutrophil	600	5
$K_{F,T}$	sites/cell	No. of IFN receptors on T cells	1000	5
$K_{F,I}$	sites/cell	No. of IFN receptors on infected cells	1300	5
MM_L	g/mol	Molecular weight of IL-6	21000	5
MM_G	g/mol	Molecular weight of GM-CSF	14000	5
MM_C	g/mol	Molecular weight of G-CSF	19600	5
MM_F	g/mol	Molecular weight of IFN-β	19000	5
		Initial conditions		
V_0	log(copies/ml)	Initial total viral load	4.5	5
S_0	10^9 cells/ml	Initial susceptible cells	0.16	5
I_0	10^9 cells/ml	Initial infected cells	0	5
R_0	10^9 cells/ml	Initial refractory cells	0	Fixed
$M_{\Phi R,0}$	10^9 cells/ml	Initial resident macrophages	2.73×10^{-5}	5
$M_{\Phi I,0}$	10^9 cells/ml	Initial inflammatory macrophages	2.9×10^{-7}	5
M_0	10^9 cells/ml	Initial monocytes	0.0004	5
M_R^*	10^9 cells/ml	Concentration of reservoir monocytes	0.0023	5
N_0	10^9 cells/ml	Initial neutrophils	0.0053	5
N_R^*	10^9 cells/ml	Concentration of reservoir neutrophils	0.0316	5
T_0	10^9 cells/ml	Initial CD8+ T cells	1.1×10^{-4}	5
$L_{U,0}$	pg/ml	Initial unbound IL-6	1.1	5
$L_{B,0}$	pg/ml	Initial bound IL-6	0	5
$G_{U,0}$	pg/ml	Initial unbound GM-CSF	2.43	5
$G_{B,0}$	pg/ml	Initial bound GM-CSF	1.6×10^{-8}	5
$C_{U,0}$	ng/ml	Initial unbound G-CSF	0.025	5
$C_{B,0}$	ng/ml	Initial bound G-CSF	6.5×10^{-10}	5
$F_{U,0}$	pg/ml	Initial unbound IFN	0.015	5
$F_{B,0}$	pg/ml	Initial bound IFN	1.1×10^{-8}	5
A_0	AU/ml	Initial antibodies	0	Fixed

Table A. Parameter values for the immunological model. Parameters added in the present study are identified by shaded grey backgrounds.

4.7 Model predictions of clinical data

We previously calibrated our immunological model to viral load data from hospitalized patients in Singapore and Germany[5], and Montréal[33]. As we modified the model structure from both Jenner et al.[5] and Gazeau et al. [4] (see Section 1), we verified that predicted viral loads remained consistent with our data (Figure E). We have previously shown that patients with moderate to severe COVID-19 can have higher viral loads[34]. That and the high heterogeneity in viral loads from individuals infected by SARS-CoV-2[35] explain the slight underprediction of our model (also present in Jenner et al.[5]), as our model prediction represents an average response to SARS-CoV-2 infection.

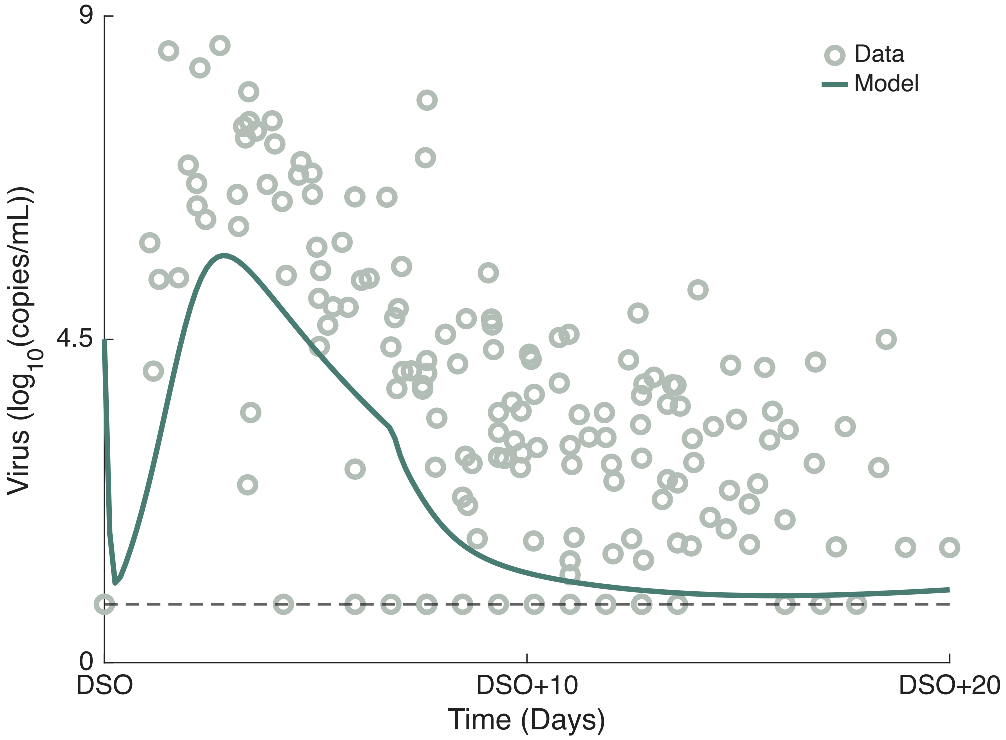

Figure E. Model prediction compared to viral loads from patients hospitalized during the first and second waves in Germany, Singapore, and Canada. Data (open grey circles) from patients hospitalized during the first and second waves used for model calibration in Jenner et al. (Singapore and Germany; original data from Goyal et al.[36]) and Gazeau et al.[33] (Canada; original data from Rébillard et al.[37]) compared to average model predicted viral load (Eq. 1 in revised Main Text). Dashed horizontal line: level of detection[33]. DSO: days from symptom onset.

4.8 Local antibody parameters sensitivity analysis

To evaluate how antibody-related parameters affect model outputs, we performed a local sensitivity analysis. For this, we varied the rates of antibody production, p_A, antibody clearance, d_A, and viral neutralization by antibodies, $\delta_{V,A}$, the half-effect antibody concentration, $\epsilon_{V,A}$, and the neutralization Hill coefficient, h_A, by $\pm$ 20% from their fixed values (see Table A). We then measured the percent change in peak concentrations in model variables during the third phase of infection. As all variables but antibodies had percent changes smaller than 20%, our results show that none of the model outputs are locally sensitive to variations in these five parameters (Figure F).

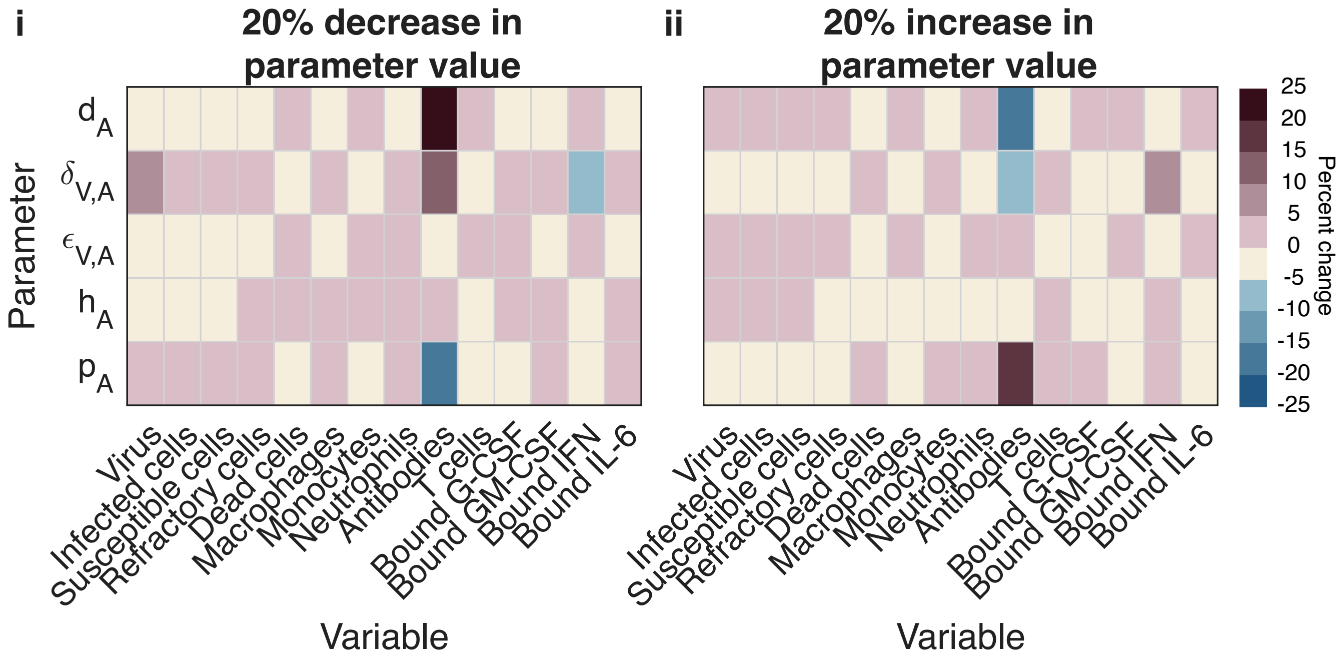

Figure F. Antibody parameter local sensitivity results. Percent change in each immunological variable (columns) resulting from **i)** a 20% decrease or **ii)** a 20% increase in antibody parameter value (rows). The percent change in each immunological variable is calculated as $(X - Y)/X \times 100$, where X and Y are the maximum value of the indicated variable in healthy and immunocompromised hosts, respectively, during the third phase of the infection; the percent change in susceptible cells is calculated with respect to the difference $S_{max} - S(t)$. Percent changes of less than 20% are considered to be insensitive to parameter value changes.

SUPPLEMENTARY REFERENCES

1 Huang, B. *et al.* Interferon response and profiling of interferon response genes in peripheral blood of vaccine-naive COVID-19 patients. *Frontiers in Immunology* **14** (2024). https://doi.org/10.3389/fimmu.2023.1315602

2 Liu, B. C., Sarhan, J. & Poltorak, A. Host-Intrinsic Interferon Status in Infection and Immunity. *Trends in Molecular Medicine* **24**, 658–668 (2018). https://doi.org/10.1016/j.molmed.2018.06.004

3 Fuchs, S. Y. Hope and Fear for Interferon: The Receptor-Centric Outlook on the Future of Interferon Therapy. *Journal of Interferon & Cytokine Research* **33**, 211–225 (2013). https://doi.org/10.1089/jir.2012.0117

4 Deng, X., Farhang-Sardroodi, S. & Craig, M. Predicting age-related determinants of heterogeneous outcomes to COVID-19 mRNA vaccines through mathematical modelling. *medRxiv*, 1–38 (2025). https://doi.org/10.1101/2025.02.14.25322308

5 Jenner, A. L. *et al.* COVID-19 virtual patient cohort suggests immune mechanisms driving disease outcomes. *PLOS Pathogens* **17**, e1009753–e1009753 (2021). https://doi.org/10.1371/journal.ppat.1009753

6 Myers, M. A. *et al.* Dynamically linking influenza virus infection kinetics, lung injury, inflammation, and disease severity. *eLife* **10** (2021). https://doi.org/10.7554/eLife.68864

7 Smith, A. M. & Perelson, A. S. Influenza A virus infection kinetics: quantitative data and models. *Wiley Interdisciplinary Reviews. Systems Biology and Medicine* **3**, 429–445 (2011). https://doi.org/10.1002/wsbm.129

8 Smith, A. P., Moquin, D. J., Bernhauerova, V. & Smith, A. M. Influenza virus infection model with density dependence supports biphasic viral decay. *Frontiers in Microbiology* **9**, 1554–1554 (2018).

9 Rochman, I., Paul, W. E. & Ben-Sasson, S. Z. IL-6 Increases Primed Cell Expansion and Survival. *The Journal of Immunology* **174**, 4761–4767 (2005). https://doi.org/10.4049/jimmunol.174.8.4761

10 Smith, T. & Cunningham-Rundles, C. Primary B-cell immunodeficiencies. *Human Immunology* **80**, 351–362 (2019). https://doi.org/10.1016/j.humimm.2018.10.015

11 Nassef Kadry Naguib Roufaiel, M., Wells, J. W. & Steptoe, R. J. Impaired T-Cell Function in B-Cell Lymphoma: A Direct Consequence of Events at the Immunological Synapse? *Frontiers in Immunology* **6** (2015). https://doi.org/10.3389/fimmu.2015.00258

12 Mancuso, S., Mattana, M., Carlisi, M., Santoro, M. & Siragusa, S. Effects of B-Cell Lymphoma on the Immune System and Immune Recovery after Treatment: The Paradigm of Targeted Therapy. *International Journal of Molecular Sciences* **23** (2022). https://doi.org/10.3390/ijms23063368

13 Sokoya, T., Steel, H. C., Nieuwoudt, M. & Rossouw, T. M. HIV as a Cause of Immune Activation and Immunosenescence. *Mediators of Inflammation* **2017**, 1–16 (2017). https://doi.org/10.1155/2017/6825493

14 Touizer, E. *et al.* Failure to seroconvert after two doses of BNT162b2 SARS-CoV-2 vaccine in a patient with uncontrolled HIV. *The Lancet HIV* **8**, e317–e318 (2021). https://doi.org/10.1016/s2352-3018(21)00099-0

15 Soumelis, V. *et al.* Depletion of circulating natural type 1 interferon-producing cells in HIV-infected AIDS patients. *Blood* **98**, 906–912 (2001). https://doi.org/10.1182/blood.V98.4.906

16 Hart, L. *et al.* Burden of chemotherapy-induced myelosuppression among patients with extensive-stage small cell lung cancer: A retrospective study from community oncology practices. *Cancer Medicine* **12**, 10020–10030 (2023). https://doi.org/10.1002/cam4.5738
17 Cassidy, T., Humphries, A. R., Craig, M. & Mackey, M. C. Characterizing Chemotherapy-Induced Neutropenia and Monocytopenia Through Mathematical Modelling. *Bulletin of Mathematical Biology* **82**, 104–104 (2020). https://doi.org/10.1007/s11538-020-00777-0
18 Holt, C. D. Overview of Immunosuppressive Therapy in Solid Organ Transplantation. *Anesthesiology Clinics* **35**, 365–380 (2017). https://doi.org/10.1016/j.anclin.2017.04.001
19 Iyaniwura, S. A. *et al.* The kinetics of SARS-CoV-2 infection based on a human challenge study. *Proceedings of the National Academy of Sciences* **121** (2024). https://doi.org/10.1073/pnas.2406303121
20 Farhang-Sardroodi, S., Deng, X., Portet, S., Arino, J. & Craig, M. Insights into B cell and antibody kinetics against SARS-CoV-2 variants using mathematical modelling. *bioRxiv* (2023). https://doi.org/10.1101/2023.11.10.566587
21 Sigal, A., Neher, R. A. & Lessells, R. J. The consequences of SARS-CoV-2 within-host persistence. *Nature Reviews Microbiology* **23**, 288–302 (2024). https://doi.org/10.1038/s41579-024-01125-y
22 Fournelle, D. *et al.* Intra-Host Evolution Analyses in an Immunosuppressed Patient Supports SARS-CoV-2 Viral Reservoir Hypothesis. *Viruses* **16** (2024). https://doi.org/10.3390/v16030342
23 Minkoff, J. M. & tenOever, B. Innate immune evasion strategies of SARS-CoV-2. *Nature Reviews Microbiology* (2023). https://doi.org/10.1038/s41579-022-00839-1
24 Beachboard, D. C. & Horner, S. M. Innate immune evasion strategies of DNA and RNA viruses. *Current Opinion in Microbiology* **32**, 113–119 (2016). https://doi.org/10.1016/j.mib.2016.05.015
25 Taylor, P. D. Allele-Frequency Change in a Class-Structured Population. *Am Nat* **135**, 95–106 (1990). https://doi.org/doi.org/10.1086/285034
26 Otto, S. P. & Day, T. *A biologist's guide to mathematical modeling in ecology and evolution*. (Princeton University Press, 2007).
27 Day, T., Kennedy, D. A., Read, A. F. & Gandon, S. Pathogen evolution during vaccination campaigns. *PLOS Biology* **20**, e3001804 (2022). https://doi.org/10.1371/journal.pbio.3001804
28 Lion, S. Class Structure, Demography, and Selection: Reproductive-Value Weighting in Nonequilibrium, Polymorphic Populations. *The American Naturalist* **191**, 620–637 (2018). https://doi.org/10.1086/696976
29 Ma, Y., Zhang, Y. & Zhu, L. Role of neutrophils in acute viral infection. *Immunity, Inflammation and Disease* **9**, 1186–1196 (2021). https://doi.org/10.1002/iid3.500
30 Watanabe, S., Alexander, M., Misharin, A. V. & Budinger, G. R. S. The role of macrophages in the resolution of inflammation. *Journal of Clinical Investigation* **129**, 2619–2628 (2019). https://doi.org/10.1172/jci124615
31 Arish, M. & Sun, J. Monocyte and macrophage function in respiratory viral infections. *Animal Diseases* **3** (2023). https://doi.org/10.1186/s44149-023-00095-7
32 McLeod, D. V. & Gandon, S. Understanding the evolution of multiple drug resistance in structured populations. *eLife* **10** (2021). https://doi.org/10.7554/eLife.65645

33 Gazeau, S. T. *et al.* Using virtual patient cohorts to uncover immune response differences in cancer and immunosuppressed COVID-19 patients. *PLOS Computational Biology* **21**, e1013170 (2025). https://doi.org/10.1371/journal.pcbi.1013170

34 Deng, X. *et al.* Plasma SARS-CoV-2 RNA elimination and RAGE kinetics distinguish COVID-19 severity. *Clinical & Translational Immunology* **12**, e1468 (2023). https://doi.org/https://doi.org/10.1002/cti2.1468

35 Owens, K., Esmaeili, S. & Schiffer, J. T. Heterogeneous SARS-CoV-2 kinetics due to variable timing and intensity of immune responses. *JCI Insight* **9** (2024). https://doi.org/10.1172/jci.insight.176286

36 Goyal, A., Cardozo-Ojeda, E. F. & Schiffer, J. T. Potency and timing of antiviral therapy as determinants of duration of SARS-CoV-2 shedding and intensity of inflammatory response. *Science Advances* **6**, eabc7112–eabc7112 (2020). https://doi.org/10.1126/sciadv.abc7112

37 Rébillard, R.-M. *et al.* Identification of SARS-CoV-2-specific immune alterations in acutely ill patients. *The Journal of Clinical Investigation* (2021). https://doi.org/10.1172/JCI145853
